# 9-cis-retinoic acid signaling in Sertoli cells regulates their immunomodulatory function to control lymphocyte physiology and Treg differentiation

**DOI:** 10.1186/s12958-024-01246-2

**Published:** 2024-06-26

**Authors:** Alicja Kamińska, Laura Pardyak, Sylwia Lustofin, Karolina Gielata, Zbigniew Arent, Agnieszka Pietsch-Fulbiszewska, Anna Hejmej

**Affiliations:** 1 Department of Endocrinology, Institute of Zoology and Biomedical Research, Faculty of Biology, Jagiellonian University, Gronostajowa 9, Krakow, 30-387 Poland; 2 Center of Experimental and Innovative Medicine, University of Agriculture in Kraków, Krakow, 30-248 Poland; 3 University Centre of Veterinary Medicine, University of Agriculture in Kraków, Krakow, 30-059 Poland

**Keywords:** Testis immune privilege, Sertoli cells, 9-cis-retinoic acid, T lymphocyte apoptosis, Treg differentiation

## Abstract

**Background:**

Testis is an immune privileged organ, which prevents the immune response against sperm antigens and inflammation. Testicular cells responsible for immune tolerance are mainly Sertoli cells, which form the blood-testis barrier and produce immunosuppressive factors. Sertoli cells prevent inflammation in the testis and maintain immune tolerance by inhibiting proliferation and inducing lymphocyte apoptosis. It has been shown that 9-cis-retinoic acid (9cRA) blocks ex vivo apoptosis of peripheral blood lymphocytes and promotes the differentiation of Treg cells in the gut. However, the role of retinoid signaling in regulating the immune privilege of the testes remains unknown.

**Objective:**

The aim of this study was to determine whether 9cRA, acting via the retinoic acid receptors (RAR) and the retinoic X receptors (RXR), controls the immunomodulatory functions of Sertoli cells by influencing the secretion of anti-inflammatory/pro-inflammatory factors, lymphocyte physiology and Treg cell differentiation.

**Methods:**

Experiments were performed using in vitro model of co-cultures of murine Sertoli cells and T lymphocytes. Agonists and antagonists of retinoic acid receptors were used to inhibit/stimulate retinoid signaling in Sertoli cells.

**Results:**

Our results have demonstrated that 9cRA inhibits the expression of immunosuppressive genes and enhances the expression of pro-inflammatory factors in Sertoli cells and lymphocytes, increases lymphocyte viability and decreases apoptosis rate. Moreover, we have found that 9cRA blocks lymphocyte apoptosis acting through both RAR and RXR and inhibiting FasL/Fas/Caspase 8 and Bax/Bcl-2/Caspase 9 pathways. Finally, we have shown that 9cRA signaling in Sertoli cells inhibits Treg differentiation.

**Conclusion:**

Collectively, our results indicate that retinoid signaling negatively regulates immunologically privileged functions of Sertoli cells, crucial for ensuring male fertility. 9cRA inhibits lymphocyte apoptosis, which can be related to the development of autoimmunity, inflammation, and, in consequence, infertility.

**Supplementary Information:**

The online version contains supplementary material available at 10.1186/s12958-024-01246-2.

## Introduction

Immune privilege implies to a specific immunological status in several places in the mammalian body where cells expressing autoantigens or foreign antigens are tolerated without provoking harmful inflammatory immune responses [1]. The mammalian testis is considered an immune privileged organ because it is able to tolerate autoantigens expressed by haploid germ cells (predominantly spermatids). These cells are produced during spermatogenesis as a result of meiotic division completed after puberty when immunocompetence is already established [2]. The immunologically privileged status of the testis has been confirmed by numerous studies on allogeneic and xenogeneic transplantation of tissues that survive for different times after implantation into the testis of small and large animals [3]. However, the testicular environment does not entirely prevent pathological conditions, such as infection, trauma, and inflammation, which might harm both steroidogenesis and spermatogenesis. Previous studies have shown that infection and inflammation of the testis is a cause of approximately 6–15% of all cases of fertility disturbances [4]. Chronic autoimmune orchitis, a frequent factor of male infertility, is thought to impair the function of the testes and epididymis through the inhibition of steroidogenesis and induction of germ cell apoptosis [5]. Therefore, it is particularly important to understand the molecular mechanisms involved in the interactions between immune system cells and testicular cells.

Research conducted in the past 25 years indicate that of all the non-immune testicular cells, Sertoli cells are main cell population responsible for immune tolerance in the testis. It was confirmed by observation that grafts transplanted into testis have been protected from rejection after most of the germ cells and testosterone producing Leydig cells were removed [6]. Furthermore, primary Sertoli cells isolated from mouse testes protected co-transplanted allogeneic and xenogeneic cells from rejection. Transplantation of Sertoli cells prolonged the survival of co-transplanted islets when they were grafted across immunological barriers, which proves that Sertoli cells are capable of retaining immunoprotective properties outside of the testis by creating an immune-privileged ectopic site [7, 8].

Sertoli cells provides structural and functional support for germ cells development. Adjacent Sertoli cells form tight junctions creating a physical blood–testis barrier (BTB) that protects the auto-immunogenic germ cells from the host’s immune system by limiting the access of germ cell antigens to interstitial immune cells and the passage of antibodies from interstitium to adluminal compartment. Blood–testis barrier was believed for many years to be critical for testicular immune privilege [9]. However, the interstitial compartment and early-stage germ cells that localize outside the BTB (spermatogonia and preleptotene spermatocytes) are also immune privilege. These observations suggested that other mechanisms are also involved in the maintenance of testicular immune privilege. Indeed, only some meiotic germ cell antigens are sequestered behind the Sertoli cell barrier while others egress from normal tubules in residual bodies during spermiation and reach the interstitium [10]. It was reported that Sertoli cells have the capacity to modulate the immune response by production and local secretion of immunomodulatory factors and cytokines, such as transforming growth factor β (TGFβ), tumor necrosis factor-α (TNFα) and its receptor TNFR1, interferons (IFN α, β, γ), interleukins (IL-1, IL-6, IL-10), galectin-1 (GAL-1), activin A, and growth factors (e.g. insulin growth factor-1, granulocyte monocyte-colony stimulating factor) [11]. These factors inhibit proliferation, regulate activation and survival of T lymphocytes present under physiological conditions in the interstitial area of the rodent testis, preventing inflammation in the testicle. In infertile patients with sperm autoimmunity, the number of lymphocytes is significantly high suggesting their involvement in testicular pathology under inflammatory conditions [12].

Several different subsets of T cells are detected in the testes, including regulatory T (Treg) cells, helper T (Th) cells, cytotoxic T (Tc) cells, and natural killer (NK) cells. It is well known that Treg cells play a relevant role in the induction of testicular immunosuppression and prevention of autoimmune diseases by suppressing other reactive T cells [5]. The Treg cell family consists of the natural CD4 + CD25 + Treg cells differentiated in thymus and the induced Treg cells generated from CD4 + CD25- precursors in periphery organs. Tregs are characterized based on the expression of the transcription factor, forkhead box P3 (FOXP3), currently the most reliable marker to identify this T cell subset [13]. Latest study demonstrated that Sertoli cells are able to induce Foxp3 expression and subsequently a regulatory phenotype in CD4 + CD25- T cells to maintain testis immunosuppression by producing TGFβ and indoleamine-2, 3-dioxygenase (IDO) [14].

Sertoli cells may also induce apoptosis of T cells, which is thought to be critical mechanism in maintaining testicular immune privilege. In the extrinsic apoptotic pathway FAS ligand (FASL) expressing Sertoli cells interacts with FAS-bearing lymphocytes, which leads to caspase activation and lymphocyte apoptosis [15]. The importance of this FAS - FASL pathway was confirmed by the study indicating that allograft rejection can be controlled by grafting of FASL-positive Sertoli cells [16, 17]. The other mechanism that mediates lymphocyte apoptosis important for the maintenance of peripheral tolerance is Bcl-2-associated X protein/B-cell lymphoma 2 (BAX/BCL-2) pathway (intrinsic mitochondrial pathway). It has been reported that the increase in BAX/BCL-2 ratio causes a caspase cascade and finally leads to the generation of an apoptotic phenotype. Notably, defects in *Bcl2* expression correlate with autoimmunity in mice and men [18].

Retinoic acids (RA), the active metabolites of vitamin A, are essential for postnatal testicular development and male fertility. In rodents, deficiency of vitamin A during spermatogenesis can cause reversible infertility within a few months. Retinoic acid signaling promotes Sertoli cell maturation at puberty by suppressing their proliferation, initiates spermatogonial differentiation and the process of meiosis [19]. Retinoids regulate cellular functions by binding to two types of intracellular receptors, retinoic acid receptors (RARs: α, β, and γ) and retinoic X receptors (RXRs: α, β, and γ), which function as ligand-dependent transcription factors. After ligand binding, homo- or heterodimers of the receptors recruit coactivators and initiate gene transcription by binding to retinoic acid response elements located within the promoters of target genes [20]. 9-cis-retinoic acid (9cRA), endogenous bioactive metabolite formed by spontaneous isomerization of all-trans RA, binds with high affinity to both RARs and RXRs in vitro [21]. It was found, that rodent Sertoli cells express both RARα and RXRβ [22]. Early investigations exhibited disruption of Sertoli cell maturation and function and spermiation failure in mice with a Sertoli cell-specific knockout of the RXRβ [23, 24]. Similarly, mice with a Sertoli cell-specific ablation of RARα showed a delay in the final phase of Sertoli cell differentiation, defects in the integrity of the Sertoli-cell barrier, progressive spermatogenic degeneration, and infertility [25]. The study showed that the overexpression of a dominant-negative form of RARα in Sertoli cells disrupts the BTB at stages VII-XII and causes the large-scale apoptosis of differentiating germ cells, which indicates that retinoids contribute to immune privilege by regulating blood-testis barrier integrity [26]. However to date, the role of retinoid signaling in regulating other Sertoli cell-dependent mechanisms involved in testicular immune privilege remains unknown.

Importantly, in the immune system retinoids blocks the ex vivo apoptosis of peripheral blood lymphocytes by regulating FASL expression and suppresses inflammatory responses [27]. Recent reports have demonstrated the role of all-trans RA in inducing Treg cells in the gut, which may be one of the mechanisms by which retinoids regulate autoimmune reactions in vivo [28]. Nevertheless, it is unclear whether retinoids may modulate testicular lymphocytes activity.

The aim of this study was to determine whether 9cRA, acting *via* RARs and RXRs controls the immunomodulatory functions of Sertoli cells by influencing the secretion of anti-inflammatory/pro-inflammatory factors, lymphocyte physiology, and the differentiation of Treg cells.

## Materials and methods

### Murine primary Sertoli cell culture and treatment

Sertoli cells were isolated from testes of ~ 16-day-old CD1 mice according to previously described protocol, which enables to obtain differentiated non-proliferating Sertoli cell of high purity [29]. Testes denuded of tunica albuginea were incubated with the mixture of collagenase Type I (0.5 mg/mL) (Sigma-Aldrich, St. Louis, MO, USA) and DNase I (200 µg/mL) (Sigma-Aldrich, St. Louis, MO, USA) for 15 min at 35 °C to disperse the seminiferous tubules. Next, tubules were washed twice in Enriched DMEM: F12 medium (VWR International, Radnor, PA, USA), layered over 5% Percoll (Sigma-Aldrich, St. Louis, MO, USA)/95% 1× Hank’s balanced salt solution (HBSS; Invitrogen, Carlsbad, CA, USA), and allowed to settle for 20 min in room temperature. The bottom part of Percoll solution was transferred and incubated for 20 min at 35 °C in the mixture of trypsin (1 mg/mL) (Thermo Fisher Scientific, Waltham, MA, USA) and DNase I (200 µg/mL). After incubation, to halt the digestion 5 mL charcoal-stripped FBS (Thermo Fisher Scientific, Waltham, MA, USA) was immediately added. The fragmented tubules were washed twice in Enriched DMEM: F12 medium (800 g, 5 min) and further digested for 30 min with the mixture of collagenase (1 mg/mL), hyaluronidase (2 mg/mL) (Sigma-Aldrich, St. Louis, MO, USA), and DNase I (50 µg/mL) to generate loose cells. The residual tubules in the suspension were filtered away from the single cell suspension with use of 100 μm cell strainer. Sertoli cells and testicular germ cells were then washed 3 times by centrifugation (800 g, 5 min) in isolation medium. Pelleted cells were plated onto 6-well plates at 1 × 10^6^ cells/cm^2^ in Enriched DMEM: F12 supplemented with serum and an antibiotic in a humidified atmosphere of 95% air and 5% CO_2_ (vol/vol) at 35^o^C. After 24 h, cultures were treated with 10% HBSS to lyse contaminating germ cells and obtain primary Sertoli cell cultures with > 90% purity. In all experiments the monolayers composed of pure Sertoli cells were used. Next, primary Sertoli cells were treated with 10^− 7^ M 9cRA (Sigma-Aldrich, St. Louis, MO, USA), 10^− 6^ M CD2665 (Tocris Bioscience, Bristol, UK), 2 × 10^− 6^ M Adapalene (Tocris Bioscience, Bristol, UK), 10^− 6^ M HX531 (Tocris Bioscience, Bristol, UK), or 10^− 5^ M Bexarotene (Tocris Bioscience, Bristol, UK) for 24 h. Control cells were incubated in the presence of the vehicle (0.01% dimethyl sulfoxide).

### Transplantation and inhibin A (INHA) expression analysis of 9cRA-treated Sertoli cells underneath kidney capsule

Twelve male CD1 mice were obtained from the Animal House, Faculty of Pharmacy, Jagiellonian University in Krakow. Animals were maintained on a 12-h light and 12-h dark cycle and received ad libitum access to standard LSM food (Agropol, Motycz, Poland) and water. Allograft transplantation was proceeded as previously described [30, 31]. Briefly, two million control or 9cRA-treated Sertoli cells (as described in 2.1) were transplanted underneath both left and right kidney capsules of healthy anesthetized mice. Negative controls constituted mice treated only with vehicle (PBS). The present study was performed according to Polish ethical and legal requirements and in compliance with the Directive 2010/63/EU on the Protection of Animals Used for Scientific Purposes. The use of the animals for the experiments was approved by 2nd Local Institutional Animal Care and Use Committee in Krakow, Poland (217/2022). The kidneys were removed from mice after 21 days and the presence of Sertoli cells was assessed by immunohistochemical analysis of inhibin A (INHA). Since INHA is not expressed in kidney cells, it was used as a marker of Sertoli cells [32]. Immunohistochemical staining was performed on paraffin sections fixed in 4% paraformaldehyde according to previously published protocol [33]. To achieve antigen retrieval, slides were immersed in 10 mM citrate buffer (pH 6.0) and heated for 5 min in a microwave oven (750 W). Sections were incubated with INHA primary antibody (PA5-13681; Thermo Fisher Scientific, Waltham, MA, USA), at 4 °C overnight followed by biotinylated secondary antibody (goat anti-rabbit IgG; 1:400; Vector, Burlingame CA, USA) for 1 h, and avidin-biotinylated horseradish peroxidase complex (ABC/HRP; 1:100; Vectastain Elite ABC Reagent, Vector Lab. Burlingame CA, USA) for 30 min. Bound antibody was visualized with 0.05% 3,3′-diaminobenzidine tetrachloride (DAB; Sigma-Aldrich, St. Louis, MO, USA). In negative control sections incubation with a primary antibody was omitted. Thereafter, sections were washed and counterstained with Mayer’s hematoxylin, dehydrated, and mounted using DPX mounting media (Sigma-Aldrich, St. Louis, MO, USA). Analyses were repeated three times. The presence of SCs and immune cell infiltration were subsequently observed with Nikon Eclipse Ni microscope at ×400 magnification (Nikon Instech Co., Ltd., Tokyo, Japan). The presence of immune cell infiltration was assessed by visual evaluation of hematoxylin-stained samples based on the morphological characteristic of immune cells (small cells with dense, dark blue nuclei; [34]).

### Isolation of murine spleen lymphocytes; 9-cis-retinoid acid-treated Sertoli cell – spleen lymphocytes co-culture

Spleen lymphocytes were isolated from healthy ~ 60-day-old mice according to previously described protocol [35]. Spleens were isolated into ice cold sterile RPMI/10% FBS medium (VWR International, Radnor, PA, USA), and gently cut into small pieces. To release the cells, spleen tissue pieces were gently pressed through the 70 μm strainer using syringe plunger. Isolated cells were centrifuged (900 g, 10 min), cell pellet was immediately disaggregated, re-suspended in 5 ml ACK (Ammonium-Chloride-Potassium) lysis buffer (Thermo Fisher Scientific, Waltham, MA, USA) and incubated on ice for 3 min to lyse red blood cells. Following the incubation, cells were washed in ice cold PBS and filtered through 70 μm cell strainer. Spleen lymphocytes (1 × 10^6^ cells/well) were co-cultured with 9cRA-pretreated Sertoli cells (1 × 10^6^ cells/well) for 48 h (1:1 ratio). Subsequently, Sertoli cells and lymphocytes were separately collected for further analyses.

### Isolation of murine CD4 + CD25- lymphocytes; 9-cis-retinoid acid-treated Sertoli cell – CD4 + CD25-lymphocytes co-culture

Single-cell suspension of CD4 + CD25- T cells was isolated and purified using EasySep™ CD4 + CD25 + T cell isolation kit (StemCell, Auburn, CA, USA) according to the manufacturer’s instructions (purity > 95%). Shortly, cells were isolated by negative selection. First, CD4 + T cells were pre-enriched using EasySep™ Mouse CD4 + T Cell Isolation Cocktail with antbodies recognizing specific cell surface markers. Then, CD25- were negatively selected using EasySep™ Mouse CD25 Regulatory T Cell Selection Cocktail, which contains antibodies recognizing CD25. The EasySep™ cocktails labeled cells with antibodies and the cells were separated using an EasySep™ magnet. Spleen CD4 + CD25- lymphocytes were co-cultured with 9cRA-treated Sertoli cells for 4 days in 1:1 ratio (1 × 10^6^ cells/well) and separately collected for further analyses.

### Lymphocyte viability and apoptosis assays

After 48 h of Sertoli cell-lymphocyte co-culture, the lymphocyte suspension was transferred to another culture plate to detect the cell viability and apoptosis by 3-(4,5- dimethylthiazol-2-yl)-2,5-diphenyltetrazolium bromide (MTT) assay and TUNEL (TdT-mediated dUTP nick end labeling) assay, respectively.

Briefly, MTT stock solution (5 mg/ml) (Thermo Fisher Scientific, Waltham, MA, USA) was added to each well and the cells were incubated at 35 °C for 6 h. A negative control was included by adding 10 µL of the MTT stock solution to medium alone. Next, 100 µL of the sodium dodecyl sulfate (SDS) – HCl solution was added to each well to dissolve the formazan. After 4-hour incubation absorbance was measured at 570 nm with microtiter plate reader (LT-4500, Labtech International Ltd., Heathfield, UK).

For TUNEL, the lymphocyte apoptosis rate was evaluated using In Situ Cell Death Detection Kit, Fluorescein (Roche, Basel, Switzerland) following the instructions of the manufacturer. Cells were counted and the percentages of TUNEL-positive lymphocytes were calculated with Leica DM IL LED microscope (Leica Microsystems Inc., Wetzlar, Germany).

### RNA isolation, reverse transcription, and quantitative RT-PCR (RT-qPCR)

Total RNA was extracted with TRIzol reagent (Life Technologies, Gaithersburg, MD, USA). Contaminating DNA and DNase was removed using TURBO DNA-free Kit (Ambion, Austin, TX, USA). The purity and quality of the RNA were examined by measuring the A260:A280 ratio (NanoDrop ND2000 Spectrophotometer, Thermo Scientific, Rocheford, IL, USA) and electrophoresis. To generate 20 µL of cDNA, High-Capacity cDNA Reverse Transcription Kit (Applied Biosystems, Carlsbad, CA, USA) was used. To estimate genomic DNA contamination of RNA samples, reactions in the absence of reverse transcriptase were carried out. RT-qPCR analyses were performed with the 10 ng cDNA templates, 0.5 µM primers (Institute of Biochemistry and Biophysics, Polish Academy of Sciences; Table 1) and SYBR Green mastermix (Applied Biosystems, Carlsbad, CA, USA) using the StepOne Real-time PCR system (Applied Biosystems, Carlsbad, CA, USA) in conditions: 55 °C for 2 min, 94 °C for 10 min, denaturation temperature 95 °C for 15 s, following by annealing temperature for 60 s to determine the cycle threshold (Ct) for quantitative measurement. Amplification efficiency was measured according to Svec et al. [36] and was between 90 and 112%. Melting curve analysis and agarose gel electrophoresis were performed to confirm amplification specificity. Negative control reactions were carried out. NormFinder was used to analyze the expression stability of commonly used reference genes and to select the most stable genes. Based on these analyses, housekeeping genes for normalizing RNA expression were selected: *Rn18s*, *B2m*, *Rpl13a*, and *Hprt1*. mRNA expressions of target genes were normalized to the geometric mean expression of the reference genes (relative quantification, RQ = 1) using the 2^−∆∆Ct^ method.

**Table 1:** Primers used in RT–qPCR analyses

Gene	Forward primer	Reverse primer
*B2m*	GGCCTGTATGCTATCCAGAA	GAAAGACCAGTCCTTGCTGA
*Bax*	CATCTTCTTCCAGATGGTGA	GTTTCATCCAGGATCGAGCAG
*Bcl2*	GAGACAGCCAGGAGAAATCA	CCTGTGGATGACTGAGTACC
*Caspase 8*	ACAATGCCCAGATTTCTCCCTAC	CAGACAGTATCCCCGAGGTTTG
*Caspase 9*	CATCCTTGTGTCCTACTCCACC	CAGCTTTTTCCGGAGGAAGT
*Fas*	GAGAATTGCTGAAGACATGACAATCC	GTAGTTTTCACTCCAGACATTGTCC
*Fasl*	GCAGAAGGAACTGGCAGAAAC	TTAAATGGGCCACACTCCTC
*Foxp3*	ATGCCCAACCCTAGGCCAGCCAAG	TGGGCCCCACTTCGCAGGTCCCGAC
*Gal1*	CGCCAGCAACCTGAATC	GTCCCATCTTCCTTGGTGTTA
*Hprt1*	GCTGACCTGCTGGATTACAT	TTGGGGCTGTACTGCTTAAC
*Ido*	GGCAAACTGGAAGAAAAAGG	CACCAGGAAATGGAACAGAATTG
*Ifng*	GGAACTGGCAAAAAGGATGGTGAC	GCTGGACCTGTGGGTTGTTGAC
*Il1a*	CTCCACCTCAATGGACAGAA	GCCGTCTTTCATTACACAGG
*Il2*	AACCTGAAACTCCCCAGGAT	CGCAGAGGTCCAAGTTCATC
*Il6*	GAACAACGATGATGCACTTGC	TCCAGGTAGCTATGGTACTCC
*Il10*	GCTATGTTGCCTGCTCTTACTG	TCTGGCTGACTGGGAAGTG
*Jnk*	GAAAACGCTGACTCAGAACAC	GCTGCACCTGTGCTAAAGGA
*Rn18s*	GTAACCCGTTGAACCCCATT	CCATCCAATCGGTAGTAGCG
*Rpl13a*	ATGACAAGAAAAAGCGGATG	CTTTTCTGCCTGTTTCCGTA
*Stat3*	GGCCCCTCGTCATCAAGA	TTTGACCAGCAACCTGACTTTAGT
*Stat5*	GTCACGCAGGACACAGAGAA	CCTCCAGAGACACCTGCTTC
*Tgfb*	TGAACCAAGGAGACGGAATACA	GGAGTTTGTTATCTTTGCTGTCACA
*Tnfr1*	GCTGGAGATGCAGAACGGGC	ACGAGGGGGCGGGATTTCTC

### Western blot analysis

Lysates were obtained by cell homogenization and sonification with RIPA buffer (Thermo Fischer Scientific, Rocheford, IL, USA) containing protease inhibitor coctail (Thermo Fisher Scientific, Rocheford, IL, USA). To determined protein concentration DC protein assay kit (Bio-Rad Laboratories, Hercules, CA, USA) was used. 30 µg of proteins were resolved by SDS-PAGE under reducing conditions followed by transfer to polyvinylidene difluoride membranes (Sigma-Aldrich, St. Louis, MO, USA). Next, 5% (wt/vol) non-fat dry milk, 0.1% (vol/vol) Tween 20 was used to block non-specific binding sites. Successively membranes were incubated with the respective primary antibody for 24 h at 4 °C (Table 2) and then with a horseradish peroxidase-conjugated secondary antibody (1:3000; Thermo Fischer Scientific, Rocheford, IL, USA) for 1 h at room temperature to detect studied proteins. Proteins were imaged with Clarity Western ECL Blotting Substrate (Bio-Rad Laboratories, Hercules, CA, USA) and chemiluminescence detection system (ChemiDoc TM XRS + System, Bio–Rad Laboratories, Hercules, CA, USA). The molecular weights of targeted proteins were assessed by reference to standard proteins (PageRulerPrestained Protein Ladder, Thermo Fisher Scientific, Rocheford, IL, USA). Finally, all membranes were stripped and reprobed with an antibody against protein loading control – β-actin (Table 2). Relative intensities of protein bands were quantified using the Image Lab software (Bio-Rad Laboratories, Hercules, CA, USA).

**Table 2:** Details of primary antibodies used for Western blot and immunofluorescence

Protein	Host species	Vendor	Cat. number	Dilution
Anti-β-actin	Mouse	Sigma-Aldrich	A2228	1:3000 (WB)
Anti-BAX	Rabbit	Sigma-Aldrich	SAB5700071	1:1000 (WB)
Anti-BCL-2	Rabbit	Bioss-Antibodies	Bs-4563R	1:500 (WB)
Anti-Caspase 8	Mouse	Cell Signaling	9746	1:1000 (WB)
Anti-Caspase 9	Rabbit	Abcam	Ab25728	1:1000 (WB)
Anti-FAS	Rabbit	Thermo Fisher	PA5-79236	1:1000 (WB)
Anti-FASL	Rabbit	Sigma-Aldrich	SAB4501538	1:2000 (WB)
Anti-FOXP3	Mouse	Sigma-Aldrich	SAB5300461	1:1000 (WB)
Anti-GAL-1	Rabbit	ABclonal	A4732	1:500 (WB); 1:100 (IF)
Anti-IDO	Rabbit	ABclonal	A12125	1:2000 (WB); 1:200 (IF)
Anti-IFN-γ	Rabbit	Biorbyt	orb214082	1:500 (WB); 1:100 (IF)
Anti-IL-1α	Rabbit	Invitrogen	P420B	1:500 (WB); 1:50 (IF)
Anti-IL-2	Rabbit	ABclonal	A16317	1:1000 (WB)
Anti-IL-6	Rabbit	Abcam	Ab290735	1:500 (WB); 1:50 (IF)
Anti-IL-10	Rabbit	Abcam	Ab9969	1:2000 (WB); 1:200 (IF)
Anti-JNK	Rabbit	Abcam	Ab179461	1:2000 (WB)
Anti-STAT3	Rabbit	ABclonal	A1192	1:500 (WB)
Anti-STAT5	Rabbit	ABclonal	A21613	1:1000 (WB)
Anti-TGFβ	Rabbit	BT Laboratory	BT-AP14912	1:2000 (WB); 1:100 (IF)
Anti-TNFR1	Rabbit	Invitrogen	PA1-40282	1:1000 (WB); 1:50 (IF)

WB-western blot; IF- immunofluorescence

### Immunofluorescence

Immunofluorescence was performed on Sertoli cells seeded on coverslips, according to a previously published protocol [37]. Briefly, cells were washed with phosphate-buffered saline (PBS) and fixed with cold methanol–acetone. Non-specific binding was blocked with normal goat serum (NGS), then primary antibodies (listed in Table 2) followed by Cy3-conjugated goat anti-rabbit IgG secondary antibody (1:200; Thermo Fischer Scientific, Rocheford, IL, USA) were used. Images were captured with epifluorescence microscope Nikon Eclipse Ni (Nikon Instech Co., Tokyo, Japan). To confirm the specifity of primary antibodies negative controls with the omission of the primary antibody were performed (not shown).

### Statistical analysis

Each data point was a mean ± SD of the results from three independent experiments. Normality and homogeneity of variance were tested with Shapiro-Wilk W-test and Levene’s test. Statistical differences in expression levels were assessed using one-way ANOVA, followed by Dunnett’s or Tukey’s post hoc comparison test or Kruskal-Wallis test. Analyses were done on raw data with Statistica 10 software (StatSoft Inc.). Data were considered statistically significant at *p < 0.05, **p < 0.01, ***p < 0.001.

## Results

### The role of 9-cis-retinoic acid in Sertoli cells and lymphocytes in the regulation of pro-inflammatory and immunosupressive genes involved in immunoregulation in the testis

**Fig. 1:**
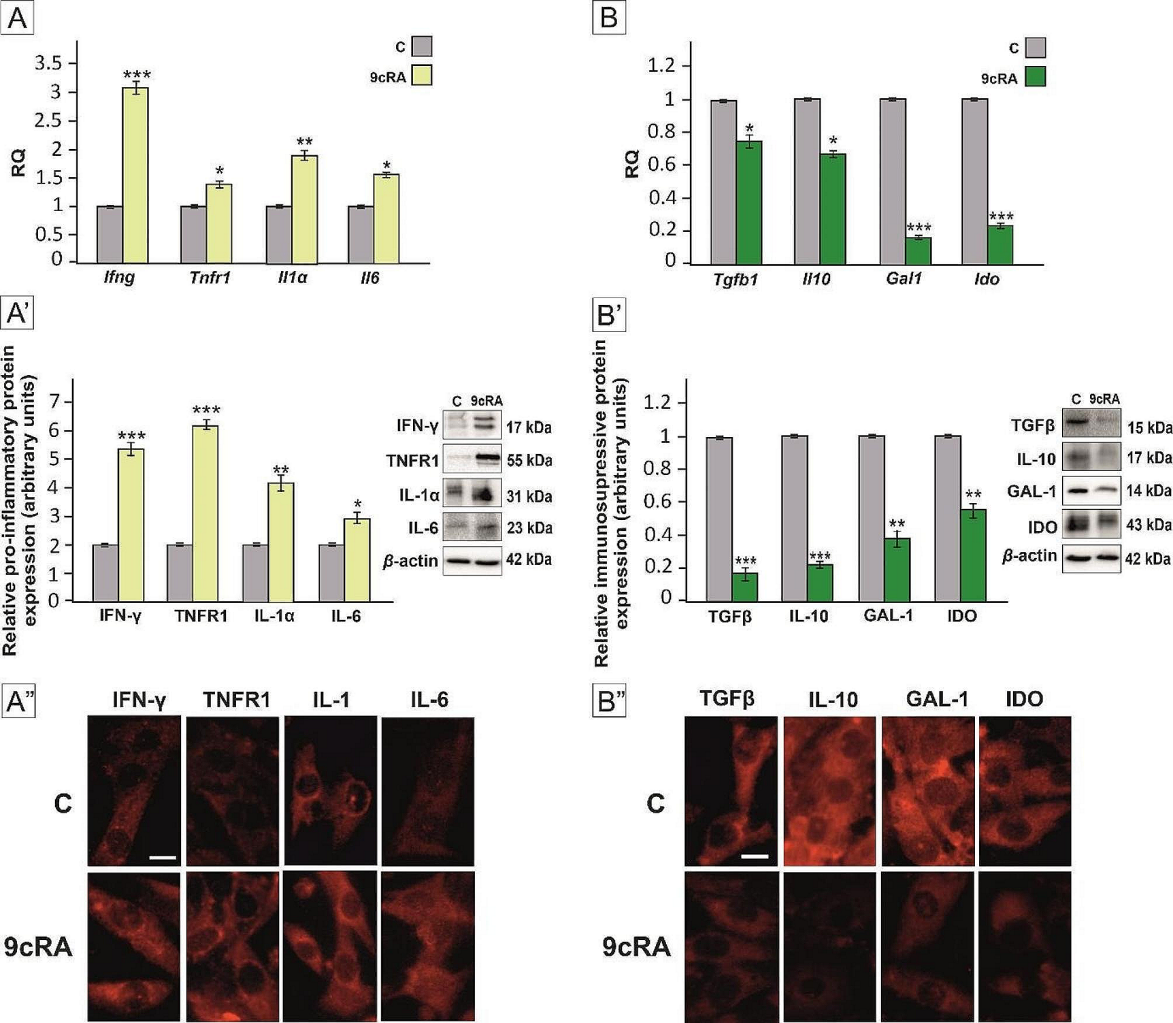
Effect of 9cRA on the expression of pro-inflammatory (**A**-**A**”) and anti-inflammatory (**B**-**B**”) genes and proteins in murine Sertoli cells. Cells were treated with a vehicle (control, C), or 10^− 7^M 9cRA for 24 h. (**A**, **B**) Relative expression of *Ifng, Tnfr1, Il1a, Il6, Tgfb1, Il10, Gal1*, and *Ido* mRNAs was determined using quantitative real-time RT–PCR analysis. The histograms are the quantitative representation of data of three independent experiments, each in triplicate. The expression of the individual genes was normalized to the mean expression of the reference genes (*Rn18s, B2m, Rpl13a*, and *Hprt1*) as an internal control (relative quantification, RQ). (**A’**, **B’**) Western blot detection of TGFβ, IL-10, GAL-1, IDO, IFN-γ, TNFR1, IL-1α, and IL-6 proteins. The histograms are the quantitative representation after densitometry of data (mean ± SD) of three independent experiments, each in triplicate. The relative level of studied protein was normalized to β-actin. The protein levels within the control group were arbitrarily set as 1. Significant differences from control values are denoted as *p < 0.05, **p < 0.01, and ***p < 0.001 (**A”**,**B”**) Subcellular localization of TGFβ, IL-10, GAL-1, IDO, IFN-γ, TNFR1, IL-1α, and IL-6 proteins in murine Sertoli cells was visualized by immunofluorescence. Scale bar = 100 μm

First, we tested the effect of 9cRA on the expression of pro-inflammatory proteins IFN-γ, TNFR1, IL-1α, and IL-6 (Fig. 1A), and anti-inflammatory proteins TGFβ, IL-10, GAL-1, and IDO in primary murine Sertoli cells (Fig. 1B). Following the exposure of Sertoli cells to 9cRA the expression of IFN-γ, TNFR1, IL-1α, and IL-6 mRNA (Fig. 1A) and protein (Fig. 1A’) was upregulated (*p* < 0.05; *p* < 0.01; *p* < 0.001). In the cytoplasm, enhanced immunofluorescence signal of IFN-γ, TNFR1, and IL-6 was observed, whereas IL-1α signal increased in perinuclear area when compared to the control (Fig. 1A”). In contrast, 9cRA administration to Sertoli cells caused decrease of the expression of TGFβ, IL-10, GAL-1, and IDO transcripts and proteins (*p* < 0.05; *p* < 0.01; *p* < 0.001) (Fig. 1B; B’). Also immunofluorescence signal was clearly reduced in cytoplasm of 9cRA-treated Sertoli cells (Fig. 1B”).

**Fig. 2:**
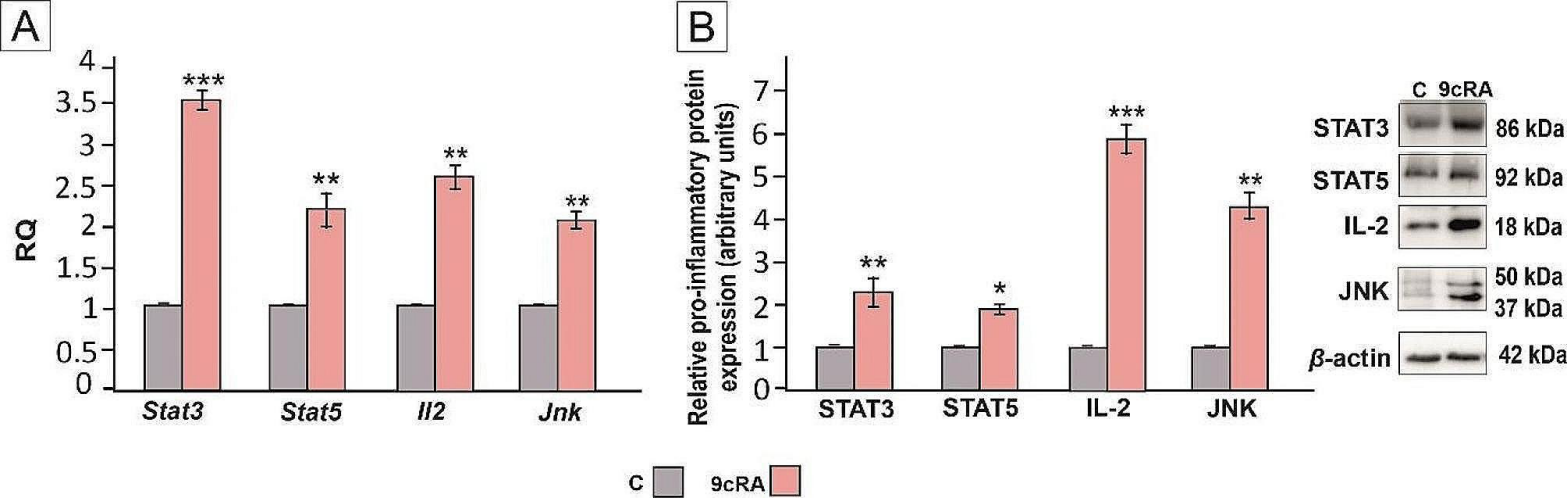
Effect of 9cRA on the expression of pro-inflammatory genes and proteins in murine lymphocytes. Cells were treated with a vehicle (control, **C**), or 10^− 7^M 9cRA for 24 h. (**A**) Relative expression of *Stat3, Stat5, Il2*, and *Jnk* mRNAs was determined using quantitative real-time RT–PCR analysis. The histograms are the quantitative representation of data of three independent experiments, each in triplicate. The expression of the individual genes was normalized to the mean expression of the reference genes (*Rn18s, B2m, Rpl13a*, and *Hprt1*) as an internal control (relative quantification, RQ). (**B**) Western blot detection of STAT3, STAT5, IL-2, and JNK proteins. The histograms are the quantitative representation after densitometry of data (mean ± SD) of three independent experiments, each in triplicate. The relative level of studied protein was normalized to β-actin. The protein levels within the control group were arbitrarily set as 1. Significant differences from control values are denoted as *p < 0.05, **p < 0.01, and ***p < 0.001

Further, we tested whether retinoid signaling activation in Sertoli cells affects lymphocyte function. For this purpose, we co-cultured 9cRA-pretreated Sertoli cells with lymphocytes and analyzed the expression of lymphocyte pro-inflammatory proteins: signal transducer and activator of transcription 3 (STAT3), signal transducer and activator of transcription 5 (STAT5), interleukin-2 (IL-2) and c-Jun N-terminal kinases 1/2/3 (JNK) (Fig. 2). Following the exposure of murine lymphocytes to 9cRA-pretreated Sertoli cells the expression of STAT3, STAT5, IL-2, and JNK mRNA (Fig. 2A) and proteins (Fig. 2B) was upregulated (*p* < 0.05; *p* < 0.01; *p* < 0.001), indicating that retinoid signaling in Sertoli cells controls the function of testicular lymphocytes.

**Fig. 3:**
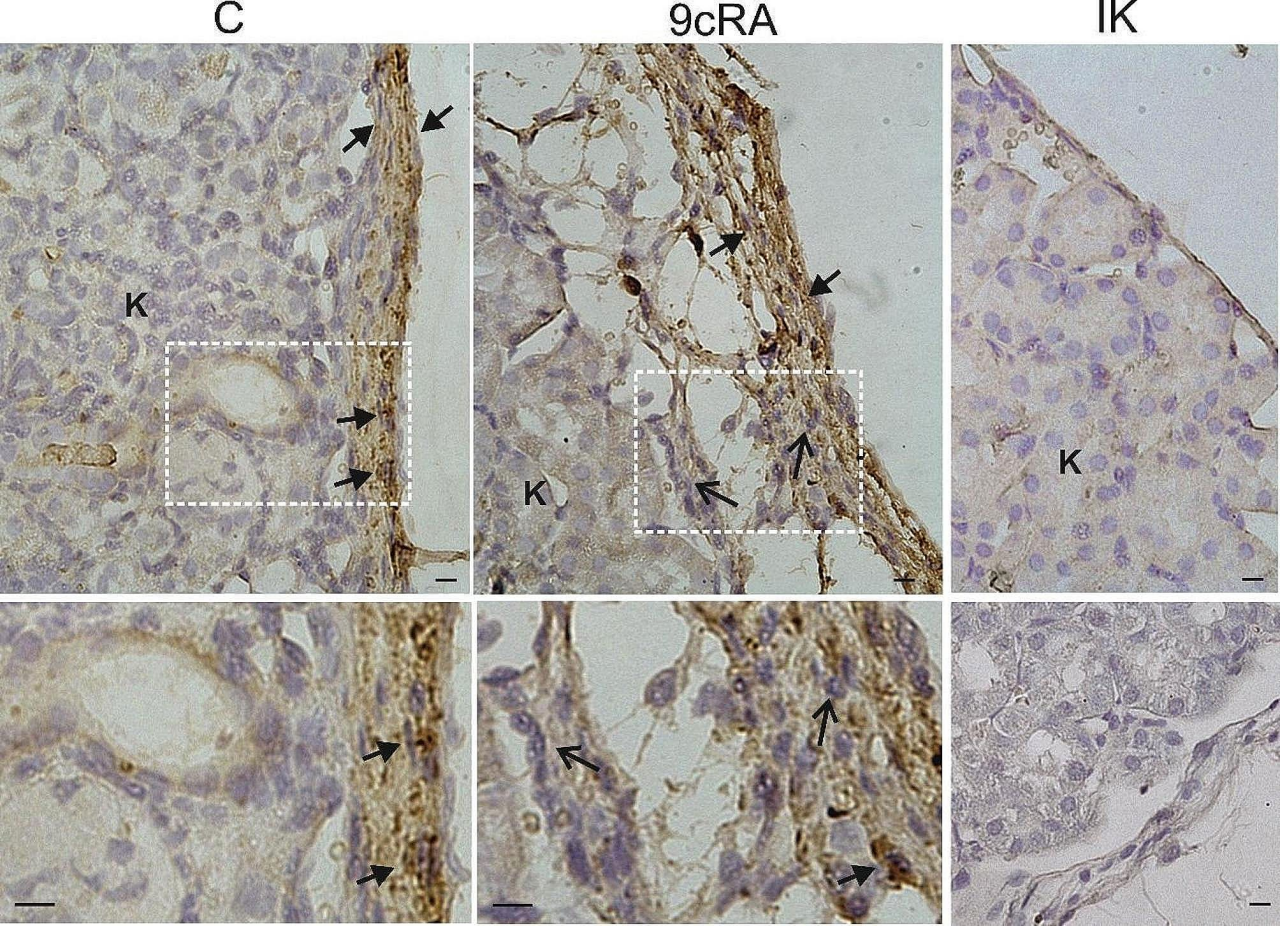
The effect of 9cRA-treated Sertoli cells transplantation underneath the kidney capsule. Immunohistochemical staining for INHA in control Sertoli cell graft (C),9cRA-treated Sertoli cell grafts (9cRA), and in the intact kidney (IK). Rectangles indicate the location of the higher magnification view (bottom images). No positive staining is observed when the primary antibody is omitted (see, right bottom section). Sections were counterstained with hematoxylin. K - kidney; arrows - Sertoli cells; open arrows - immune cells. Scale bars represent 20 μm

In order to evaluate the immunologically privileged capacity of 9cRA-treated Sertoli cells, Sertoli cells were transplanted into kidney capsule as allografts (Fig. 3). Clusters of INHA-positive Sertoli cells occurred both in the control and 9cRA-treated group. No positive signal was found in the intact kidneys of vehicle-treated mice. The presence of clear immune cell infiltration was visible only after transplantation of 9cRA-treated Sertoli cells. This confirms the results of in vitro studies and indicates the proinflammatory role of 9cRA signaling in Sertoli cells.

### The role of 9-cis-retinoic acid signaling in Sertoli cells in the control of viability and apoptosis of lymphocytes

**Fig. 4:**
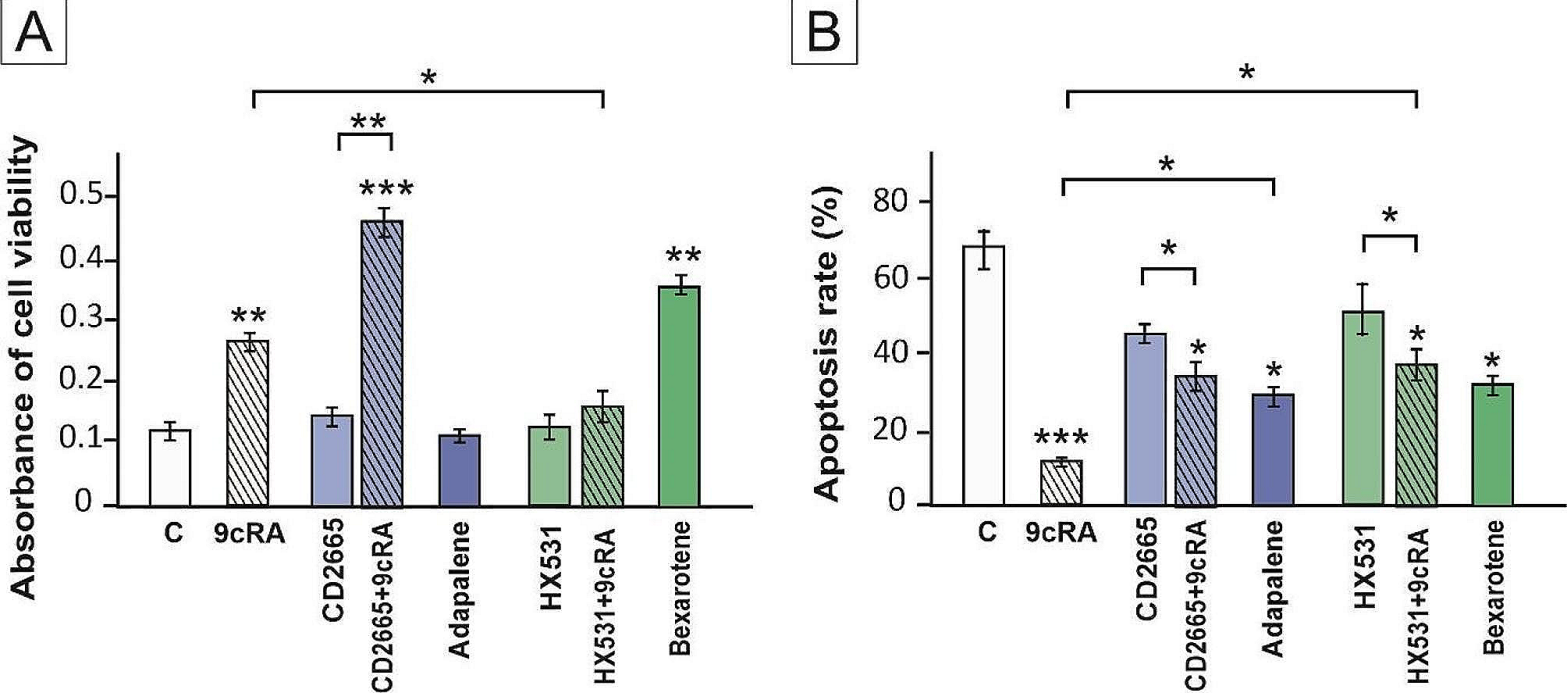
Effect of retinoid signaling on murine T lymphocytes viability (MTT assay; **A**) and apoptosis (TUNEL assay; **B**). Sertoli cells were pre-treated with a vehicle (control, C), 10^− 7 ^M 9cRA, 10^− 6 ^M CD2665, 9cRA + CD2665, 2 × 10^− 6 ^M Adapalene, 10^− 6 ^M HX531, 9cRA + HX531, or 10^− 5 ^M Bexarotene for 24 h. Spleen lymphocytes were co-cultured with 9cRA-pretreated Sertoli cells for 48 h. Bars represent mean ± SEM. Significant differences from control values are denoted as *p < 0.05, **p < 0.01, and ***p < 0.001

The co-culture model of murine Sertoli cells and lymphocytes allowed to analyze the impact of 9cRA-pretreated Sertoli cells on the lymphocyte viability and apoptosis. Based on MTT analysis we found that 9cRA significantly enhanced lymphocyte viability compared with the control group (*p* < 0.01) (Fig. 4A). RXR agonist (Bexarotene) increased lymphocyte viability to the level comparable to 9cRA-treated cells (*p* < 0.01), while RXR antagonist (HX531) completely abolished the effect of 9cRA on cell viability (*p* < 0.05). In contrast, neither RAR agonist (Adapalene) nor RAR antagonist (CD2665) had an effect on basal and 9cRA-stimulated lymphocyte viability level. This indicates that RXR-dependent signaling is involved in the regulation of lymphocytes viability.

The results of TUNEL assay revealed that 9cRA as well as RAR and RXR agonists significantly reduced the apoptosis rate of lymphocytes (*p* < 0.001) (Fig. 4B). The effect of 9cRA was partly suppressed by both RAR and RXR antagonists (*p* < 0.05), indicating that 9cRA acting through both RAR and RXR decreases the apoptosis rate in lymphocytes co-cultured with murine Sertoli cells (Fig. 4B).

### Effects of 9-cis-retinoic acid signaling in Sertoli cells on the FASL/FAS/Caspase 8 and BAX/BCL-2/Caspase 9 pathways

To explain the mechanism of the induction of lymphocyte apoptosis by 9cRA-pretreated Sertoli cells, the expression of proteins involved in both intrinsic and extrinsic apoptotic pathways was analyzed in 9cRA-pretreated Sertoli cells co-cultured with lymphocytes.

**Fig. 5:**
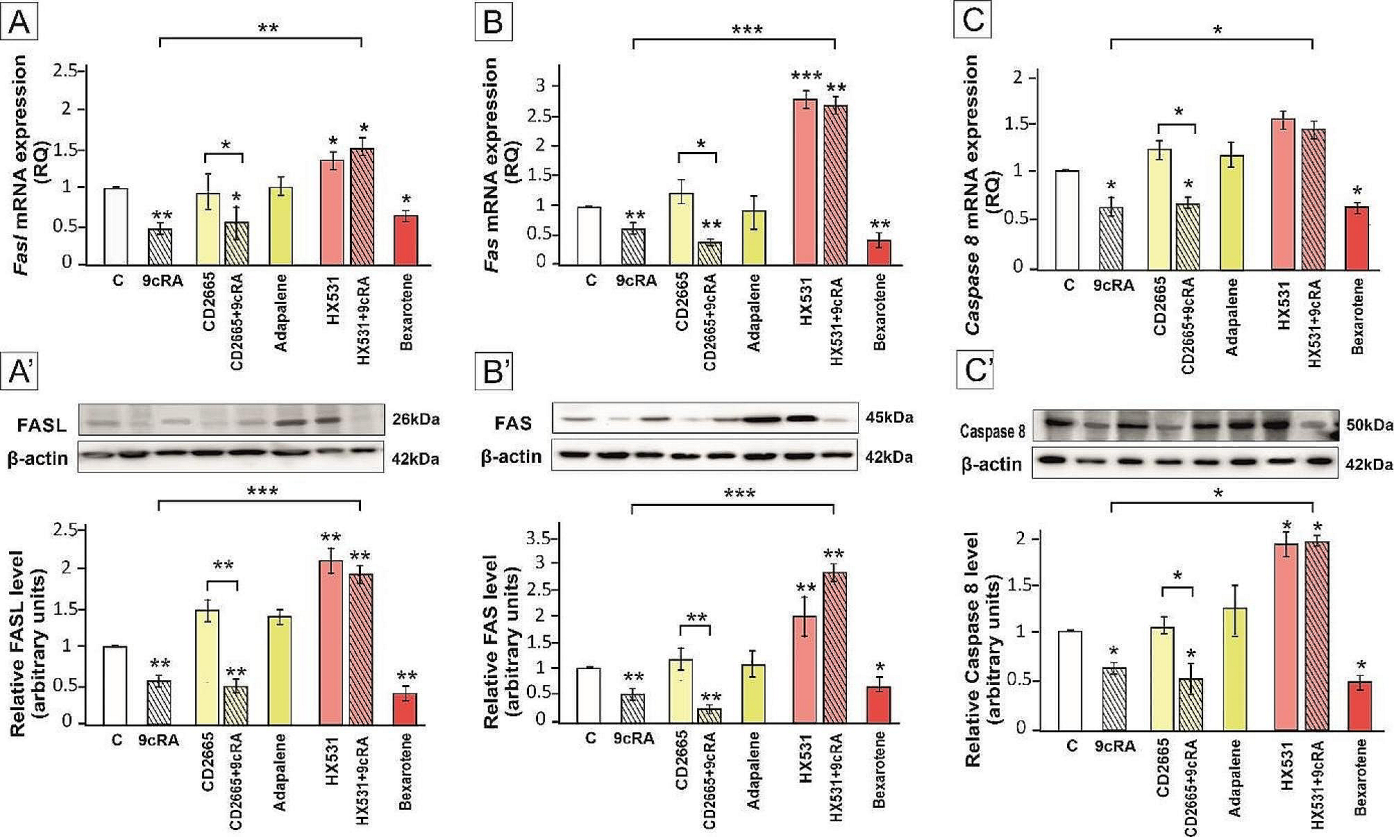
The expression of FASL (**A**, **A’**) in Sertoli cells, FAS (**B**, **B’**) and Caspase 8 (**C**, **C’**) in lymphocytes co-cultured with Sertoli cells pre-treated with 9cRA or RAR/RXR agonists or antagonists. Sertoli cells were pre-treated with a vehicle (control, C), 10^− 7 ^M 9cRA, 10^− 6 ^M CD2665, 9cRA + CD2665, 2 × 10^− 6 ^M Adapalene, 10^− 6 ^M HX531, 9cRA + HX531, or 10^− 5 ^M Bexarotene for 24 h. Spleen lymphocytes were co-cultured with 9cRA-treated Sertoli cells for 48 h. (**A**, **B**, **C**) Relative expression of *Fasl, Fas*, and *Caspase8* mRNAs was determined using quantitative real-time RT–PCR analysis. The histograms are the quantitative representation of data of three independent experiments, each in triplicate. The expression of the individual genes was normalized to the mean expression of the reference genes (*Rn18s*, *B2m*, *Rpl13a*, and *Hprt1*) as an internal control (relative quantification, RQ). (**A’**, **B’**, **C’**) Western blot detection of FASL, FAS, and Caspase8 proteins. The histograms are the quantitative representation after densitometry of data (mean ± SD) of three independent experiments, each in triplicate. The relative level of studied protein was normalized to β-actin. The protein levels within the control group were arbitrarily set as 1. Significant differences from control values are denoted as *p < 0.05, **p < 0.01, and ***p < 0.001

The expression of FASL, FAS, and Caspase 8 was reduced after incubation with 9cRA and this effect was abrogated only by RXR antagonist (*p* < 0.05; *p* < 0.01; *p* < 0.001). Moreover, only RXR agonist suppressed proteins expression (*p* < 0.05; *p* < 0.01). RAR agonist and antagonist had no effect on the expression of analyzed proteins. This indicates that primarily RXR is involved in the regulation of FASL/FAS/Caspase 8 pathway in Sertoli cells and lymphocytes (Fig. 5A-C’).

**Fig. 6:**
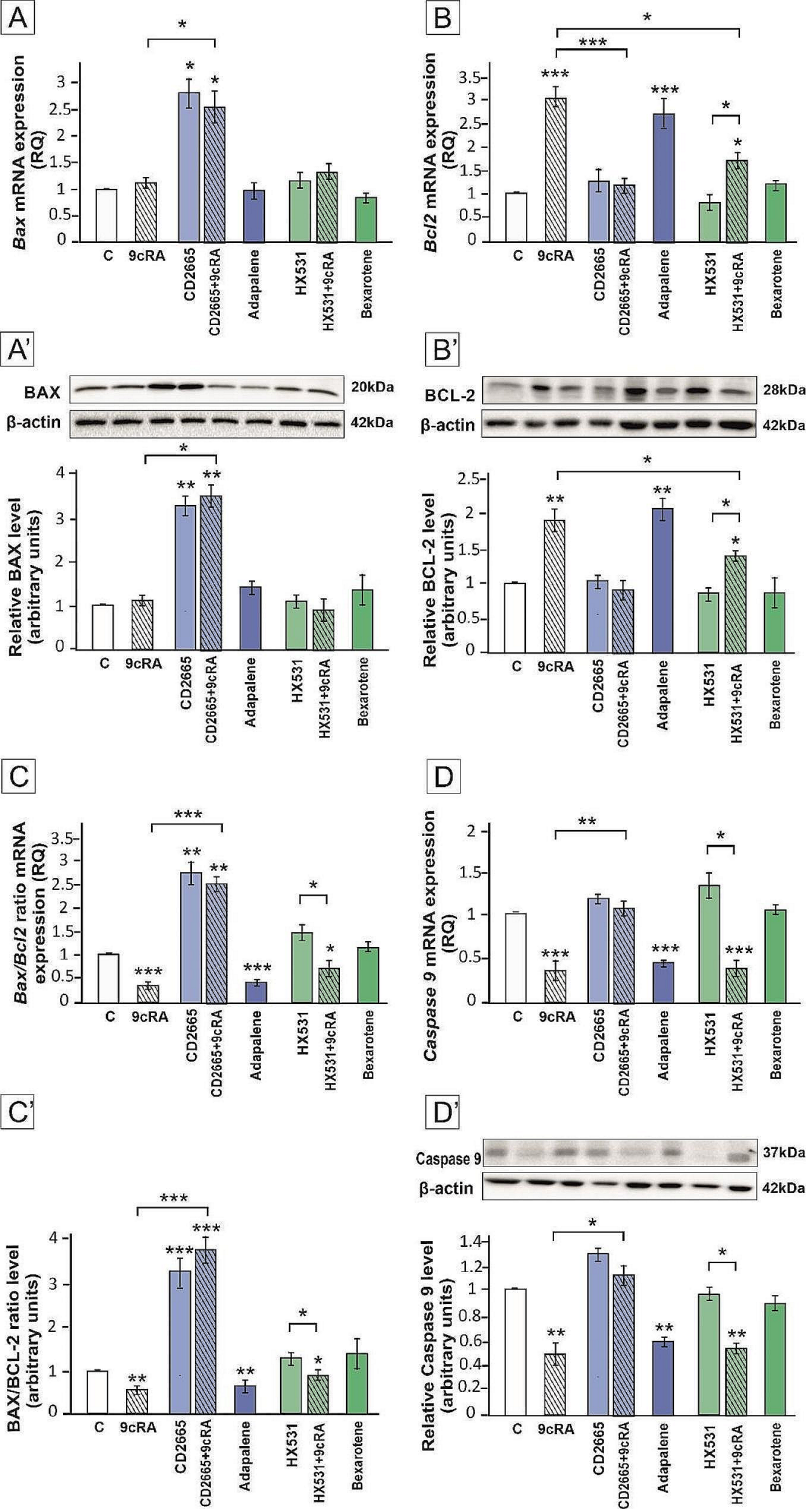
The expression of BAX (**A**, **A**’), BCL-2 (**B**, **B’**), and Caspase 9 (**D**, **D**’) in lymphocytes co-cultured with Sertoli cells pre-treated with 9cRA or RAR/RXR agonists or antagonists. Sertoli cells were pre-treated with a vehicle (control, **C**), 10^− 7 ^M 9cRA, 10^− 6 ^M CD2665, 9cRA + CD2665, 2 × 10^− 6 ^M Adapalene, 10^− 6 ^M HX531, 9cRA + HX531, or 10^− 5 ^M Bexarotene for 24 h. Spleen lymphocytes were co-cultured with 9cRA-treated Sertoli cells for 48 h. **(A**, **B, D**) Relative expression of *Bax, Bcl2*, and *Caspase9* mRNAs was determined using quantitative real-time RT–PCR analysis. The histograms are the quantitative representation of data of three independent experiments, each in triplicate. The expression of the individual genes was normalized to the mean expression of the reference genes (*Rn18s*, *B2m*, *Rpl13a*, and *Hprt1*) as an internal control (relative quantification, RQ). (**A’, B**’, **D’**) Western blot detection of BAX, BCL-2, and Caspase 9 proteins. The histograms are the quantitative representation after densitometry of data (mean ± SD) of three independent experiments, each in triplicate. The relative level of studied protein was normalized to β-actin. The calculated ratio of BAX/BCL-2 mRNA (**C**) or protein (**C’**) expression. The protein levels within the control group were arbitrarily set as 1. Significant differences from control values are denoted as *p < 0.05, **p < 0.01, and ***p < 0.001

Upregulation of BCL-2 expression and downregulation of BAX/BCL-2 ratio and Caspase 9 expression were found in murine lymphocytes after 9cRA treatment when compared to the control (p < 0.01; p < 0.001) (Fig. 6B-D’). The effect of 9cRA on BAX, BCL-2, and Caspase 9 expression was completely abolished only by RAR antagonist (*p* < 0.05; *p* < 0.01; *p* < 0.001) (Fig. 6A-D’). What is more, only exposure to the RAR agonist altered the expression of studied proteins (*p* < 0.01; *p* < 0.001). Thus, RAR seems to play a dominant role in the control of BAX/BCL-2/Caspase 9 pathway in lymphocytes.

### Effects of 9-cis-retinoic acid signaling in Sertoli cells on the Treg cell differentiation

Latest study demonstrated that TGFβ is able to induce *Foxp3* expression and subsequently a regulatory phenotype in CD4 + CD25- murine T cells [38]. Herein, we demonstrated that treatment with 9cRA decreased the expression of TGFβ in Sertoli cells (see subsection 3.1). Therefore, indirect effect of 9cRA signaling in Sertoli cells on Treg differentiation may be proposed. To test such a possibility, we isolated CD4 + CD25- T cells from spleen, co-cultured them with 9cRA-treated Sertoli cells, and analyzed FOXP3 expression.

**Fig. 7:**
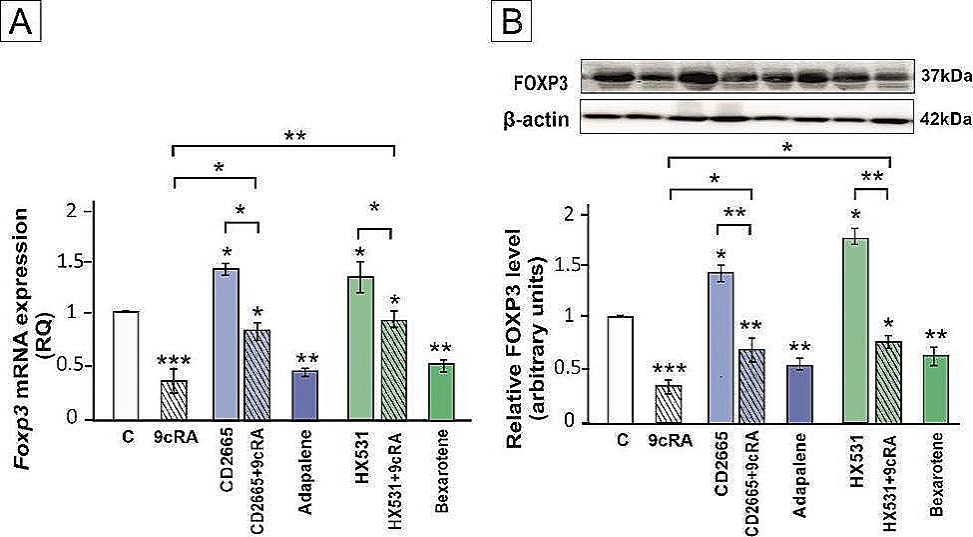
The expression of FOXP3 in CD4 + CD25- lymphocytes co-cultured with Sertoli cells pre-treated with 9cRA or RAR/RXR agonists or antagonists. Sertoli cells were pre-treated with a vehicle (control, C), 10^− 7 ^M 9cRA, 10^− 6 ^M CD2665, 9cRA + CD2665, 2 × 10^− 6 ^M Adapalene, 10^− 6 ^M HX531, 9cRA + HX531, or 10^− 5 ^M Bexarotene for 24 h. CD4 + CD25- lymphocytes were co-cultured with 9cRA-treated Sertoli cells for 4 days. (**A**) Relative expression of *Foxp3* mRNAs was determined using quantitative real-time RT–PCR analysis. The histograms are the quantitative representation of data of three independent experiments, each in triplicate. The expression of the individual genes was normalized to the mean expression of the reference genes (*Rn18s*, *B2m*, *Rpl13a*, and *Hprt1*) as an internal control (relative quantification, RQ). (**B**) Western blot detection of FOXP3 protein. The histograms are the quantitative representation after densitometry of data (mean ± SD) of three independent experiments, each in triplicate. The relative level of studied protein was normalized to β-actin. The protein levels within the control group were arbitrarily set as 1. Significant differences from control values are denoted as *p < 0.05, **p < 0.01, and ***p < 0.001

Both RT-qPCR and western blot revealed that 9cRA as well as RAR and RXR agonists downregulate the FOXP3 expression (*p* < 0.05; *p* < 0.01; *p* < 0.001). In T cells, RAR and RXR antagonists partly abrogated the effect of 9cRA on FOXP3 expression, which suggests that 9cRA signaling through both receptors affects Treg cell differentiation (*p* < 0.05; *p* < 0.01) (Fig. 7A-B).

## Discussion

**Fig. 8:**
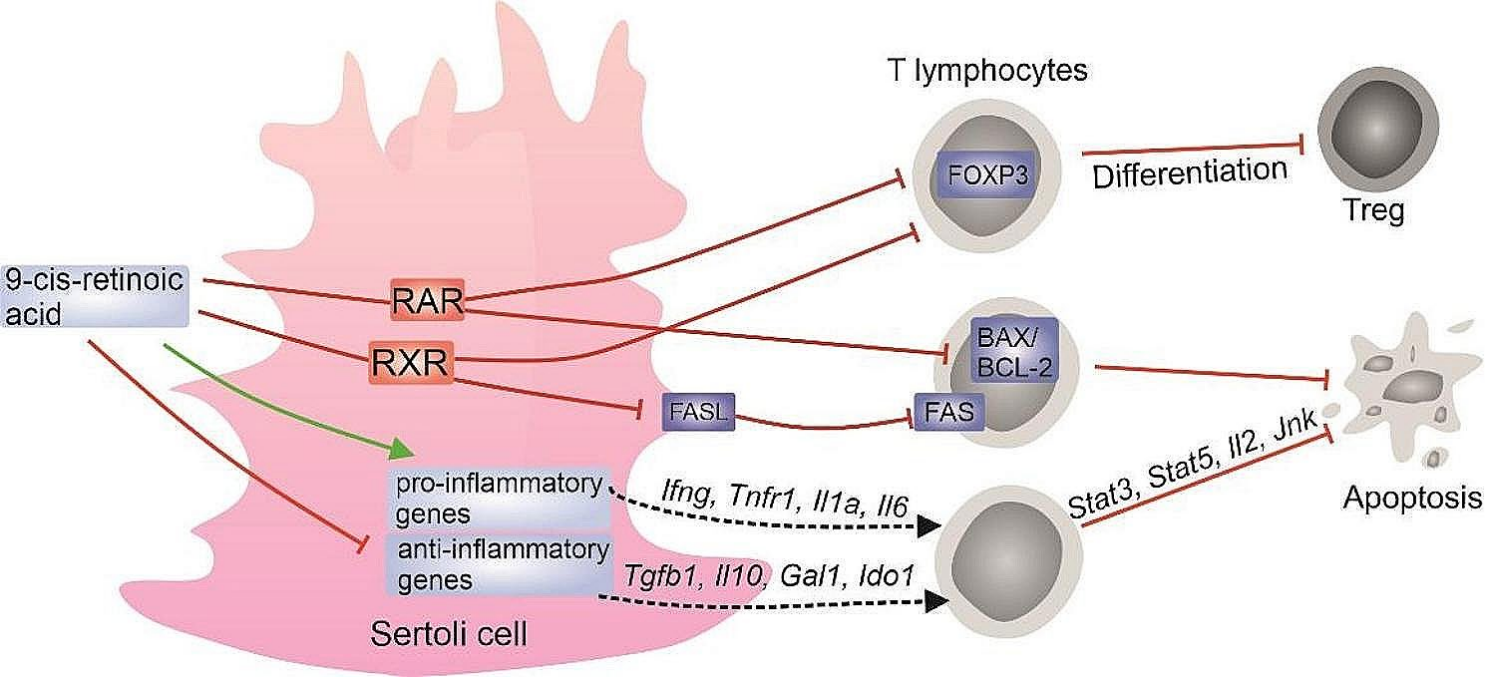
Schematic illustration summarizing main results obtained in the present study

We have presented here novel data demonstrating a link between retinoid signaling in Sertoli cells and testis immune privilege. Our results provided evidence that 9cRA signaling in Sertoli cells increases the expression of pro-inflammatory genes, while reducing the expression of anti-inflammatory genes, affects lymphocyte viability, blocks their apoptotic death and inhibits Treg differentiation, which can be related to the development of autoimmunity and inflammation (Fig. 8).

Previous studies demonstrated that 9cRA signaling induces production of pro-inflammatory cytokines by dendritic cells, promotes the differentiation of effector T cells and the formation of lymphoid structures [39, 40]. In parallel to the activation of the innate immune response, RA can also promote human dendritic cells to induce production of T cells expressing anti-inflammatory *Il10* gene, controlling inflammation and tissue homeostasis [41]. Therefore, the outcome of retinoid signaling depends on the cell type or its physiological state.

Herein, we revealed for the first time that exposure of murine primary Sertoli cells to 9cRA leads to upregulation of *Ifng, Tnfr1, Il1a*, and *Il6* genes. It is well established that elevated expression of these genes is closely related with the induction and progression of the inflammation in the testis. Elhija and coworkers [42] observed over-expression of IL-6 in testicular homogenates of mature and immature mice after systemic inflammation. Moreover, intratesticular injection of IL-6 induced inflammation and damage of seminiferous tubules. Interestingly, rat Sertoli cells cultured in the presence of IL-6 exhibited a redistribution of tight junction proteins [43]. In rat in vivo model of experimental autoimmune orchitis (EAO), an increase in the number of TNFR1 and IL-6 positive cells has been linked to progression of the disease [44, 45]. In EAO rat testis involvement of IFN-γ in inflammatory infiltration was also detected [46]. Equally important, an increase in IL-1 expression during testis inflammation and its role in neutrophil recruitment from the testicular vasculature have also been demonstrated [47]. Moreover, a few studies proved that IL-1, IL6, and TNFα stimulate each other’s production in an amplification loop, progressing T lymphocyte activation in many tissues [48, 49]. Notably, TNFα was able to increase IL-1α and IL-6 production in rat Sertoli cells [50].

On account of these data, increase in IFN-γ, TNFR1, IL-1α, and IL-6 expression in Sertoli cells following 9cRA allows us to consider 9cRA as a factor promoting pro-inflammmatory phenotype of murine Sertoli cells. This is confirmed by the observation that in murine Sertoli cells 9cRA downregulates the expression of genes involved in maintaining immune tolerance in the testis: *Tgfb, Il10, Gal1*, and *Ido*. It was previously found that TGFβ protected islet β-cell grafts after co-transplantation with Sertoli cells [51]. Moreover, overexpression of IL-10 in the testis significantly reduced inflammation in EAO model [52], as well as administration of exogenous GAL-1 suppressed the clinical signs of EAO and testicular inflammation, and promoted the differentiation of tolerogenic dendritic cells and Tregs [53, 54]. However, a pro-inflammatory role of GAL-1 in Sertoli cells was also reported [55]. Finally, IDO expressed by rat Sertoli exerts immunosuppressive effect, modulating inflammatory response to germ cell antigens [56].

To further confirm that 9cRA signaling in Sertoli cells may disrupt testicular immune privilege, we examined the expression of pro-inflammatory proteins in lymphocytes co-cultured with 9cRA-stimulated Sertoli cells and found upregulation of IL-2, STAT3, STAT5, and JNK expression. It was previously reported that T lymphocytes responsible for the induction of EAO secrete IL-2 and inhibition of IL-2/STAT5 signaling is essential for the development of FOXP3 + Tregs [44, 57, 58]. Activation of STAT3 is associated with the progression of autoimmunological aggression [59]. Notably, IL-6 strongly regulates STAT3 activation [60], which is in agreement with our results, showing an increase in the expression of both proteins in Sertoli cells. JNK regulates the proliferation, maturation, and activity of T cells and synthesis of pro-inflammatory cytokines IL-2, IL-6, and TNF-α. Several studies demonstrated the importance of JNK pathway in inflammatory disorders such as rheumatoid arthritis and atherosclerosis [61, 62]. In the light of the above-mentioned data, enhanced expression of IL-2, STAT3, STAT5, and JNK in response to 9cRA detected in our study may contribute to impaired T lymphocyte physiology and disruption of testis immune privilege. An in vivo model was used to confirm the role of 9cRA in the control of immune privilege. In the present study, lymphocyte infiltrations were observed underneath the kidney capsule 20 days after transplantation of 9cRA-treated Sertoli cells, which indicates that 9cRA weakened the ability of Sertoli cells to prevent the aggression of lymphocytes.

Testis as immune-privileged site can prolong the survival of allografts and xenografts [30, 63]. The capability of Sertoli cells to inhibit T lymphocyte proliferation, confirmed in cell culture experiments, could be highly related to the graft survival [64]. Therefore, to explain the effects of retinoid-dependent changes in Sertoli cell secretory activity on lymphocyte fate, we examined whether 9cRA signaling in Sertoli cells regulates T lymphocyte proliferation and apoptosis.

We observed increase in viability of lymphocytes co-cultured with 9cRA-pretreated Sertoli cells, which indicates that retinoid signaling activation limits the ability of Sertoli cells to prevent the proliferation of lymphocytes. Our results showed that prolonged lymphocyte survival is dependent on 9cRA action through RXRs in Sertoli cells. Previously, only direct relationship between RXRα expression in T lymphocytes and cell cycle progression and cell proliferation was demonstrated [65]. Although (based on our results) RARs activation in Sertoli cells does not affect lymphocyte viability, activation of RARs signaling directly in T cells or neural stem cells was found to affect proliferation of these cells [66, 67].

We further demonstrated that 9cRA, acting in Sertoli cells *via* both RARs and RXRs, inhibited lymphocyte apoptotic cell death, for the first time providing evidence for Sertoli cell-mediated effect of retinoids on lymphocytes apoptosis. So far, only the direct effect of retinoids on cell apoptosis has been detected in T cell hybridomas, thymocytes and peripheral blood lymphocytes [68–70]. This effect was described as mediated through both RARs and RXRs and involved in blockade of FASL expression [70, 71].

In our co-culture experiments, mRNA and protein expressions of FASL in RA-treated Sertoli cells as well as FAS and Caspase 8 in lymphocytes were significantly suppressed, which indicates that Sertoli cell - lymphocyte FAS/FASL/Caspase8 system was disturbed by 9cRA. Of note, earlier studies revealed inhibitory effect of TNFα on FASL expression in Sertoli cells and demonstrated a link between Sertoli cell expression of TGFβ1 and FASL and lymphocyte apoptosis [72, 73]. Therefore, it is likely that TNFα and TGFβ1, which have been upregulated in our study after exposure of Sertoli cells to 9cRA, contribute to indirect inhibition of lymphocyte apoptosis by retinoids. It is worth to mentioning, however, that in fibroblasts 9cRA exerts stimulatory effect on the expression of FASL, resulting in the induction of Jurkat T cells death [74].

Based on the results presented herein, the regulation of FASL/FAS/Caspase8 pathway in murine Sertoli cell - lymphocytes co-culture by 9cRA is dependent on RXRs. In contrast, RARs activation in Sertoli cells downregulates intrinsic apoptotic pathway (reducing BAX/ BCL-2 ratio and Caspase 9) in this model. It was previously reported that Sertoli cells prevent germ cell apoptosis *via* contact dependent or paracrine cell-to-cell interactions by upregulating BCL-2 [75]. Notably, direct inhibitory effects of RARγ activation on this pathway has been reported to limit cell apoptosis in adenocarcinoma cells [76].

Collectively, these results indicate the inhibitory effect of 9cRA on lymphocyte apoptosis mediated through activation of both RARs and RXRs in Sertoli cells, leads to suppresion of the FASL/FAS/Caspase8 and BAX/BCL-2/Caspase 9 pathways, which may reduce testis immune privilege.

Lastly, we provided evidence that retinoid signaling in Sertoli cells can affect Treg differentiation. The ability of Sertoli cells to induce the generation of Tregs was reported previously in diabetic mouse model [77]. Although, the exact mechanism has not been fully understood, earlier in vitro studies showed that mouse Sertoli cells induce Tregs from naive T cells after exposure to IFN-γ [78]. It has been proposed that Sertoli cells can promote Tregs differentiation (within or outside the testis) either directly by secreting regulatory factors such as soluble JAGGED1, TFG-β, IL-10, and IDO, or indirectly through activation of other immunotolerant cells [79, 80]. In view of these data, inhibitory effect of 9cRA/RAR/RXR signaling on the expression of Treg marker FOXP3 detected in our study may result from the downregulation of TFG- β, IL-10, and IDO found in Sertoli cells after 9cRA treatment.

Finally, it is important to note that RXRs control gene expression by forming heterodimers not only with RARs, but also with other nuclear receptors (such as peroxisome proliferator-activated receptors (PPAR), pregnane X receptor (PXR), liver X receptor (LXR), farnesoid X receptor (FXR)). Thus, the observed effect of RXRs in the regulation of immune privilege in the testis may be additionally related to other signaling pathways, which requires further investigation.

## Conclusion

It is widely known that vitamin A is necessary for the proper course of spermatogenesis and maintaining male fertility. However, our work has shown for the first time that the activation of retinoid signaling in Sertoli cells by 9cRA may limit their immunosuppressive functions. It is likely, therefore, that excessive activation of retinoid receptors negatively affects Sertoli cells immunomodulatory properties, which can potentially lead to inflammation and impaired fertility. Thus, our findings point to the importance of maintaining the appropriate level of retinoic acid signaling in the testis.

Despite many years of research knowledge regarding the interaction of the immune system cells and the male reproductive system in the process of creating and maintaining an immunologically privileged environment in the male gonad of mammals is still incomplete. Nevertheless, our present results shed light on the mechanisms involved in the indirect effect of Sertoli cells on the function of lymphocytes and the induction of Treg differentiation, which is particularly important to understand the molecular basis of the interactions between immune and testicular cells.

### Electronic supplementary material

Below is the link to the electronic supplementary material.

Supplementary Material 1

## Data Availability

The data that support the findings of this study are available on request from the corresponding author.

## Abbreviations

9cRA: 9-cis-Retinoic Acid
ACK: Ammonium-Chloride-Potassium
Bax: Bcl-2-associated x protein
Bcl-2: B-cell lymphoma 2
BTB: Blood-Testis Barrier
DMEM: Dulbecco’s Modified Eagle Medium
FasL: Fas Ligand
FBS: Fetal Bovine Serum
FoxP3: Forkhead box P3
FXR: Farnesoid X Receptor
GAL-1: Galectin-1
HBSS: Hank’s Balanced Salt Solution
IDO: Iindoleamine-2, 3-Dioxygenase
IFN α,β: Interferonαβ
IL-1: Interleukin 1
IL-2: Interleukin 2
IL-6: Interleukin 6
IL-10: Interleukin 10
INHA: Inhibin A
JNK: c-Jun N-terminal Kinases 1/2/3
LXR: Liver X Receptor
NHS: Normal Horse Serum
NK: Natural Killer T cells
PPAR: Peroxisome Proliferator-Activated Receptors
PXR: Pregnane X Receptor
RAR: Retinoid Acid Receptors
RXR: Retinoid X Receptors
STAT3: Signal Transducer and Activator of Transcription 3
STAT5: Signal Transducer and Activator of Transcription 5
Tc: Cytotoxic T cells
TGFβ: Transforming Growth Factor β
Th: Helper T cells
TNFα: Tumor Necrosis Factor-α
TNFR1: Tumor Necrosis Factor-α Receptor
Treg: Regulatory T cells
