# Supplementary material for: 9-cis-retinoic acid signaling in Sertoli cells regulates their immunomodulatory function to control lymphocyte physiology and Treg differentiation
Source: Reprod Biol Endocrinol. 2024 Jun 26;22:75. doi: 10.1186/s12958-024-01246-2 (PMC11202360; doi:10.1186/s12958-024-01246-2)

Figure 1.

IFNγ

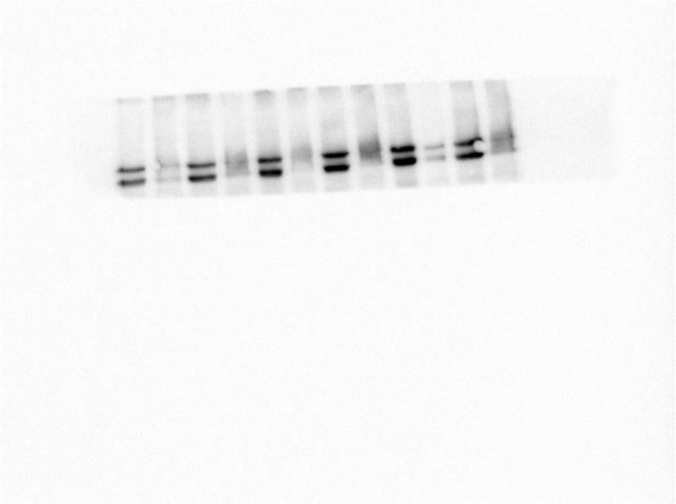

TNFR1

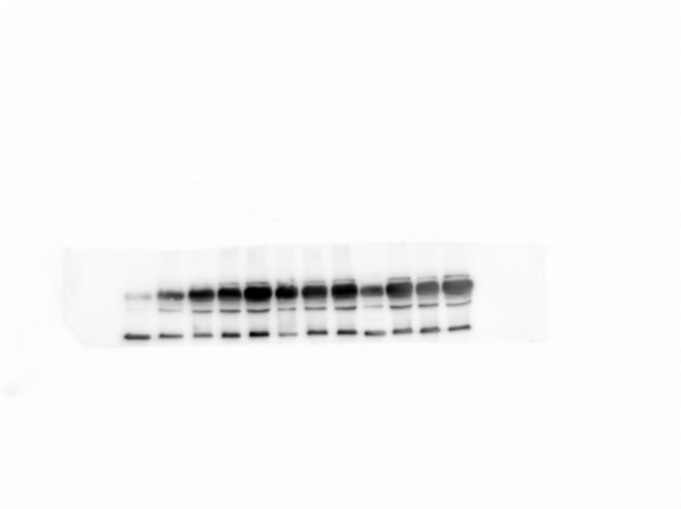

IL-1α

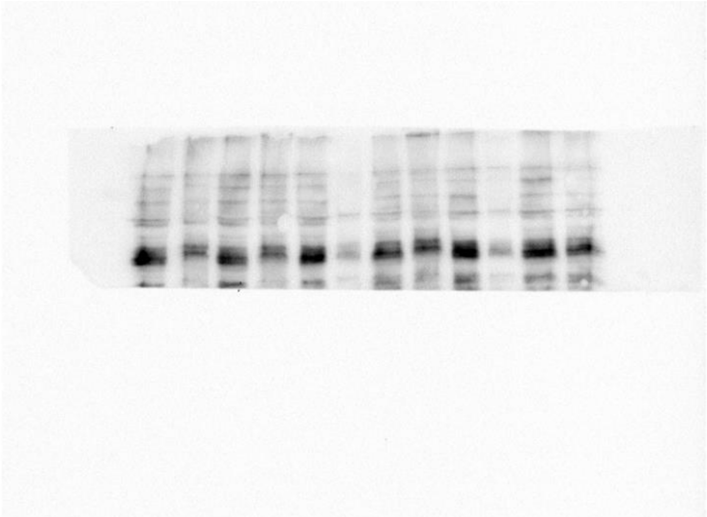

IL-6

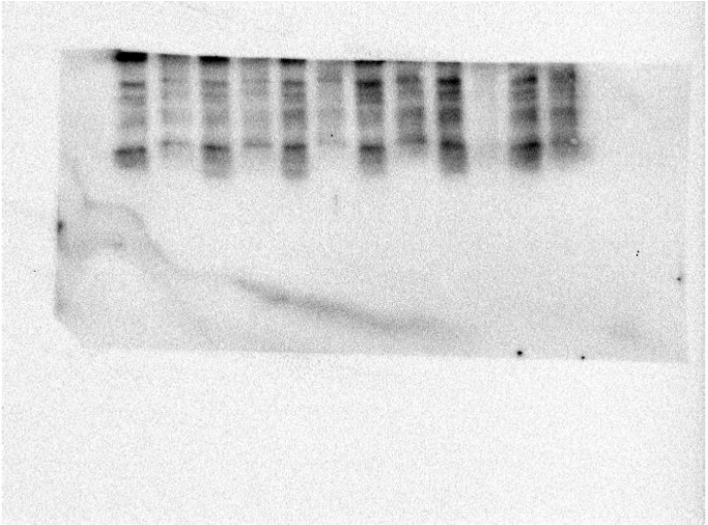

Β-actin

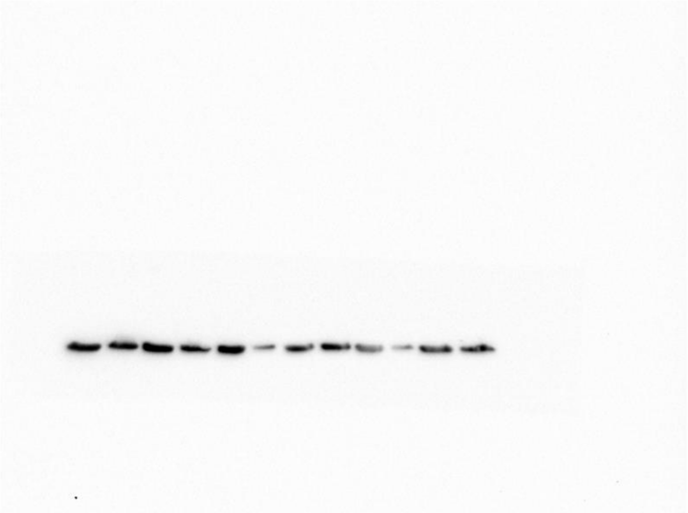

TGFβ

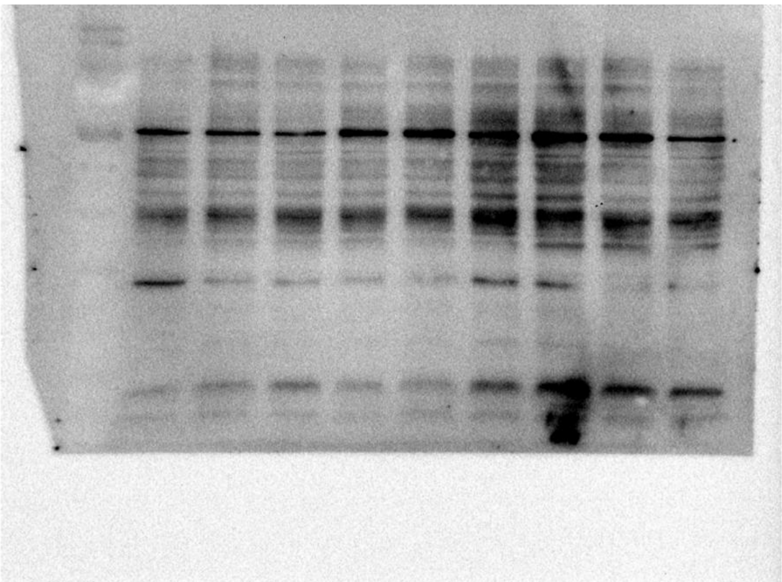

IL-10

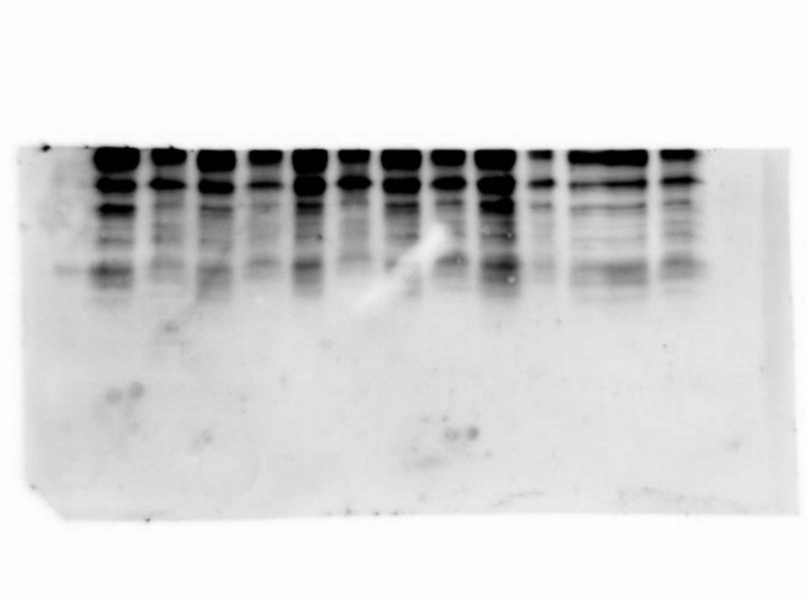

GAL-1

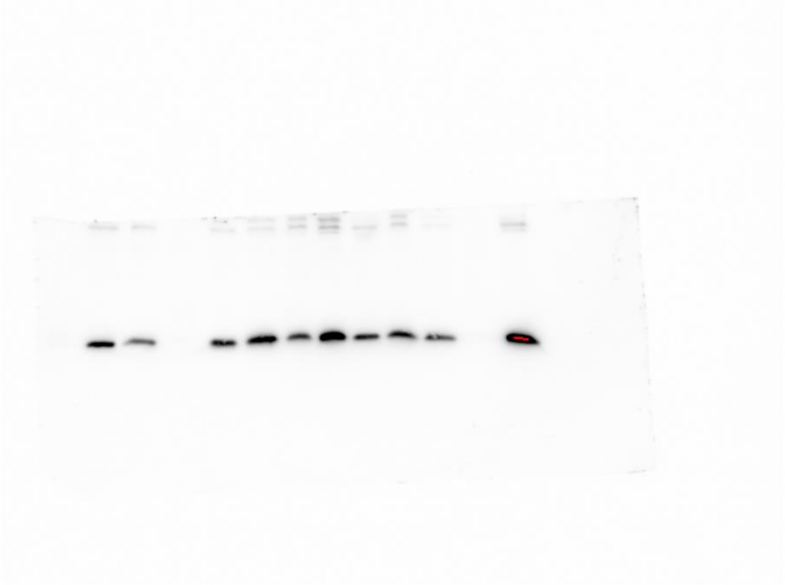

IDO

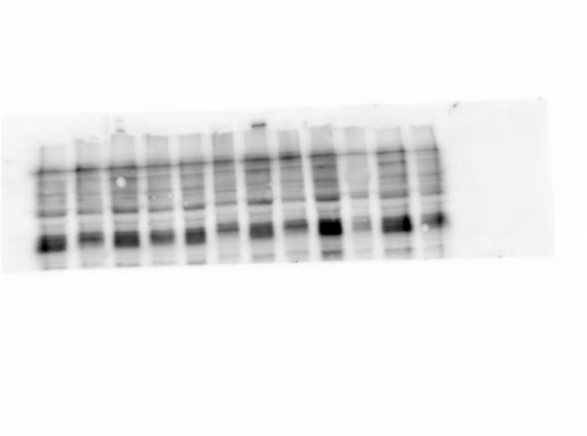

B-actin

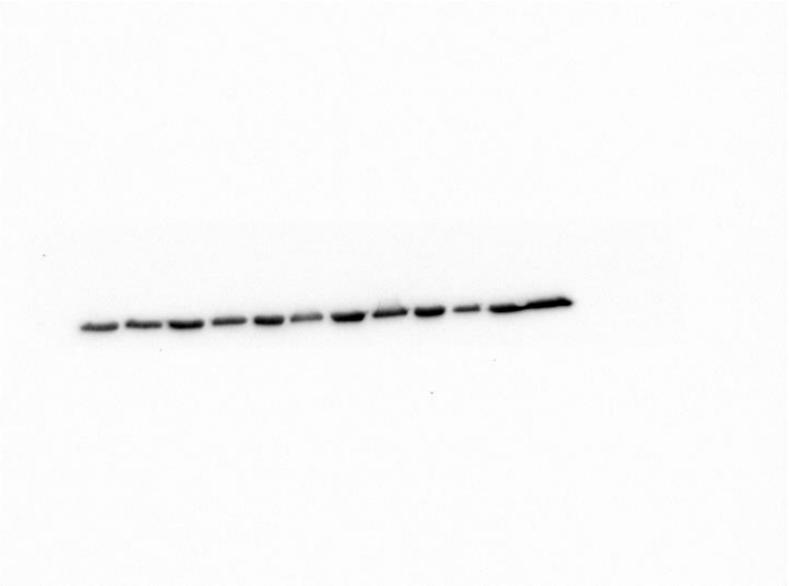

Figure 2.

STAT3

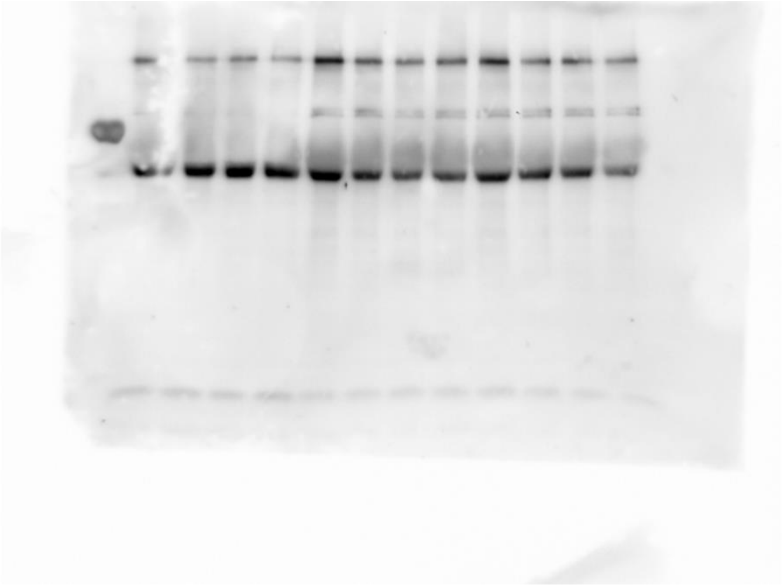

STAT5

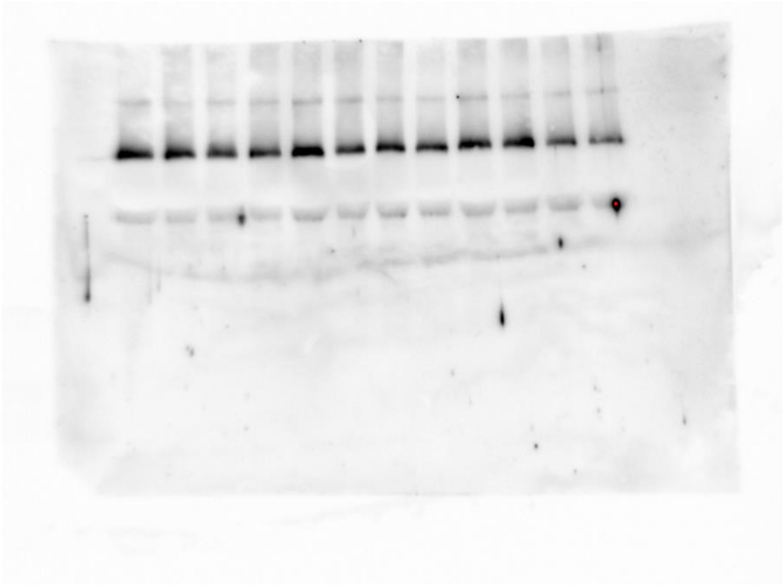

IL-2

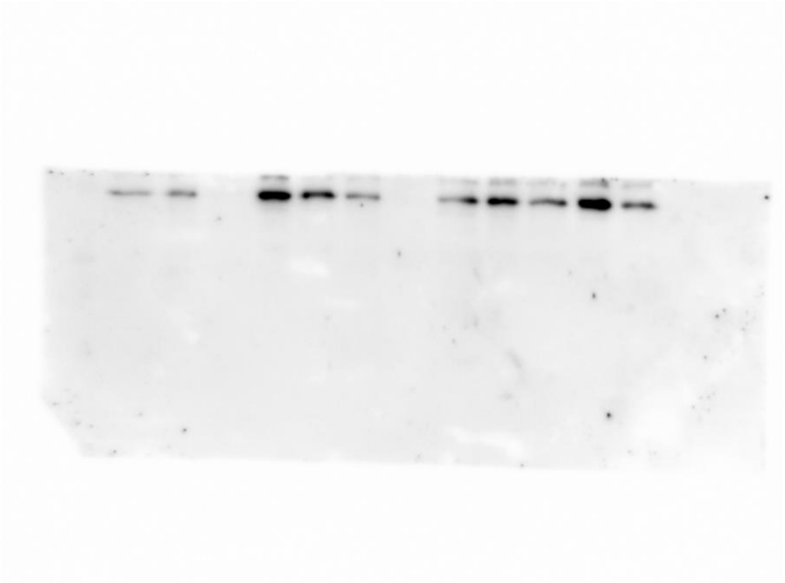

JNK

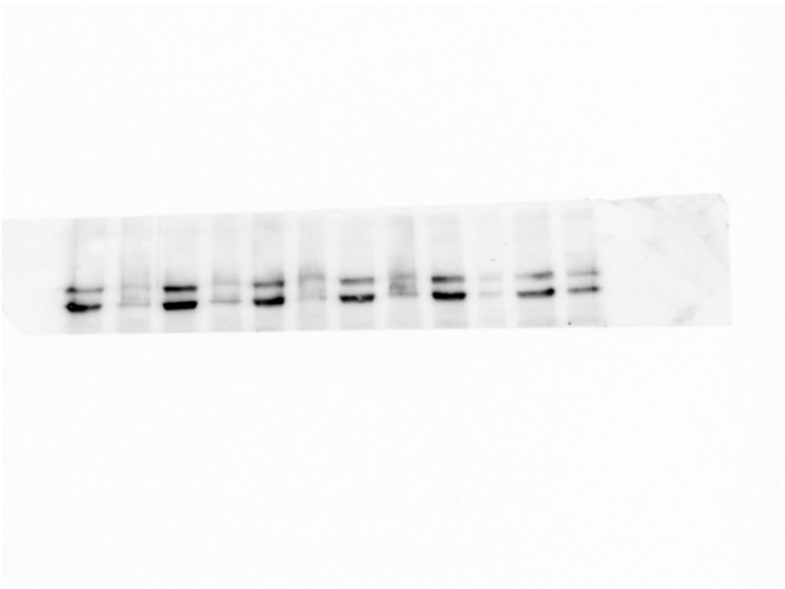

B-actin

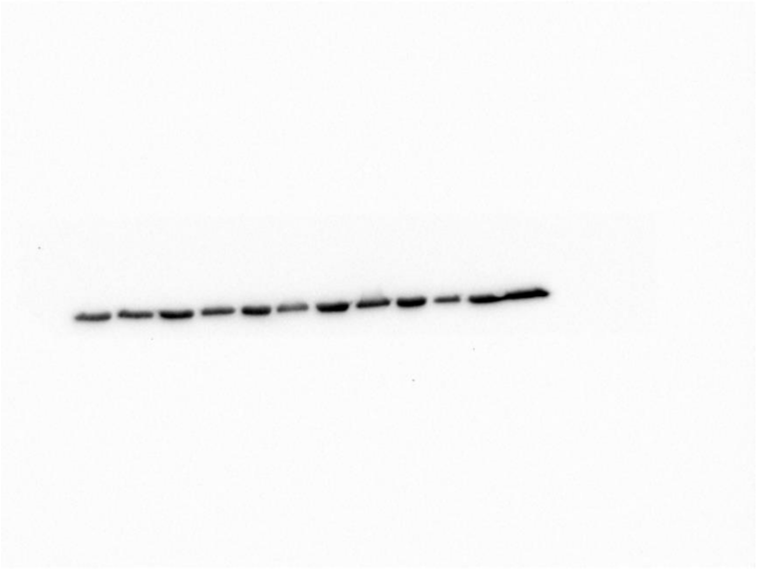

Figure 4.

FASL

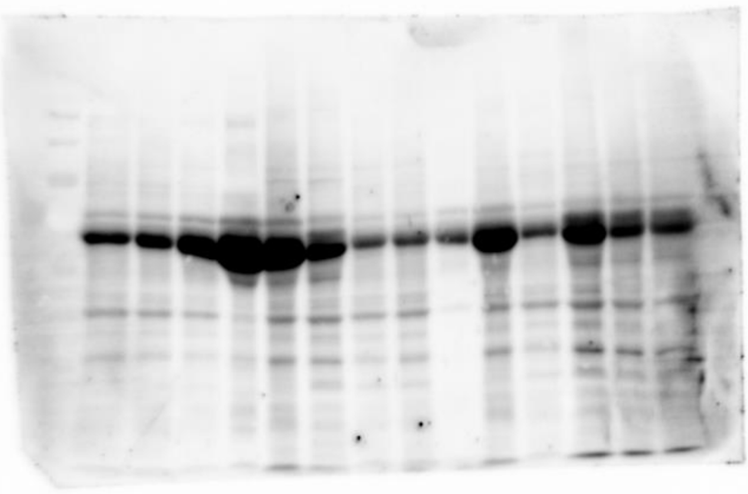

B-actin

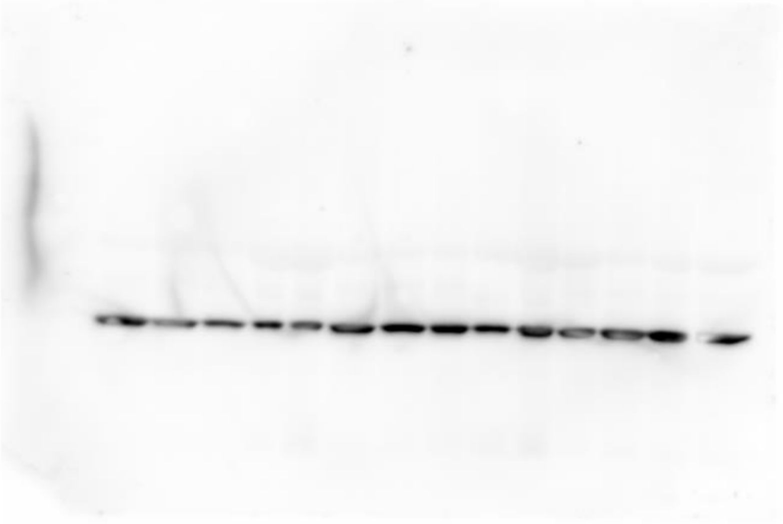

FAS

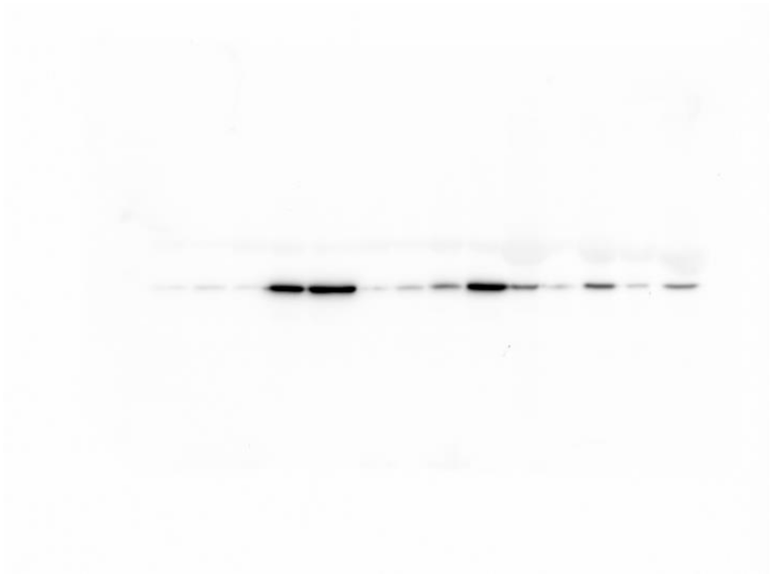

B-actin

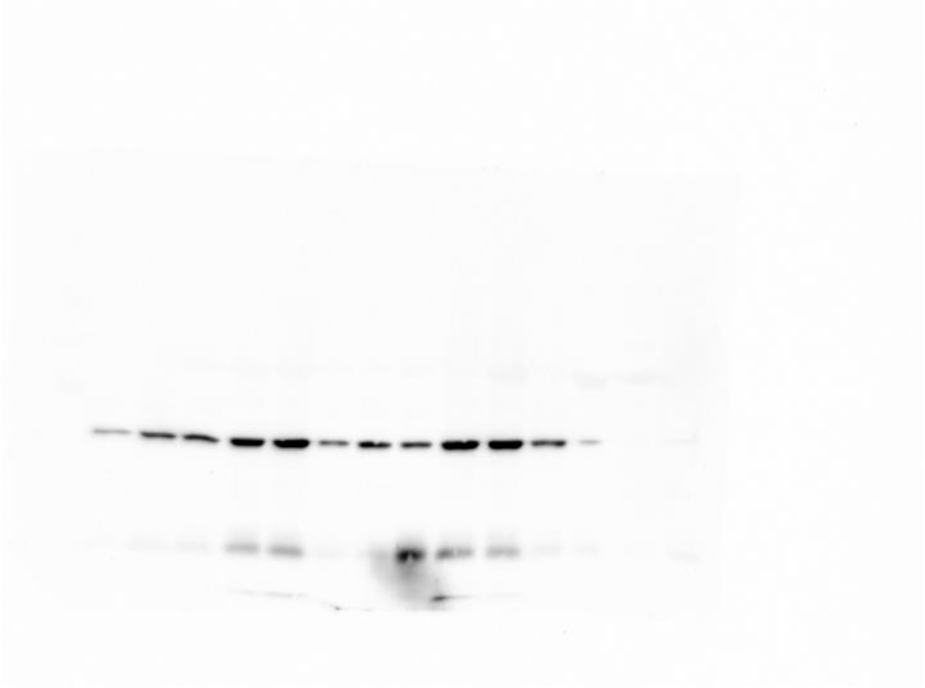

Caspase 8

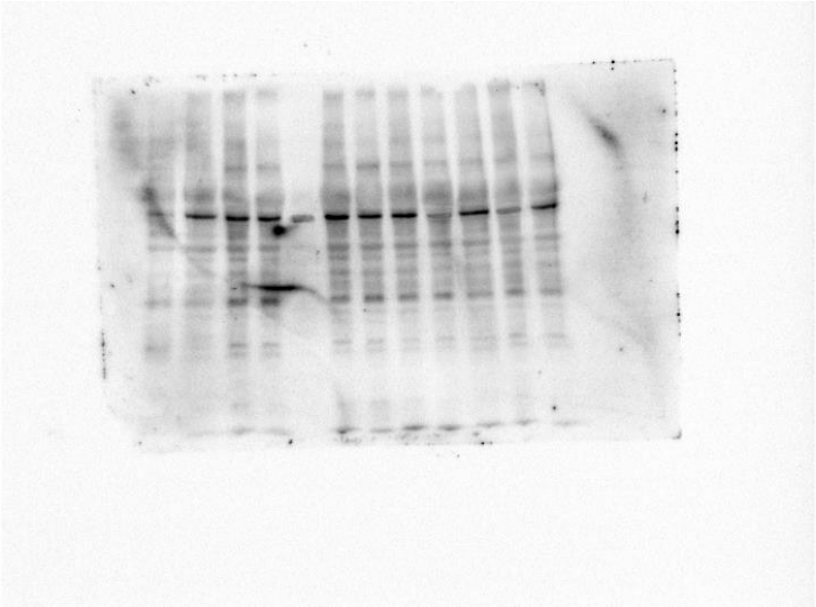

B-actin

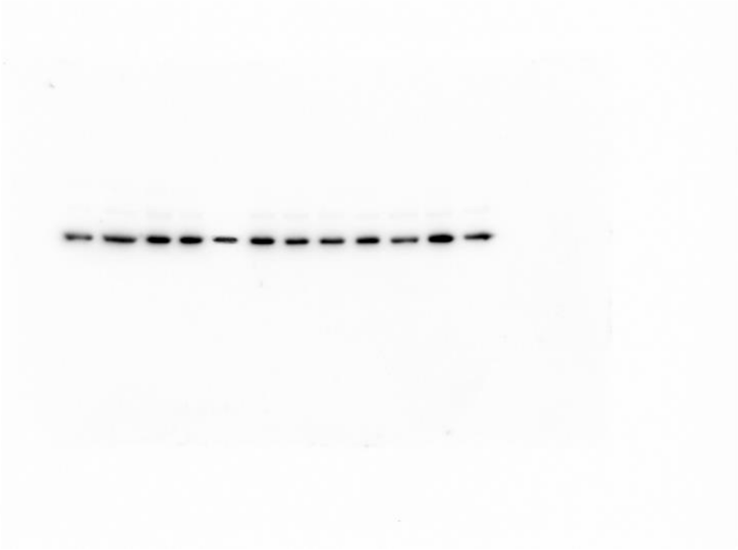

Figure 5.

BAX

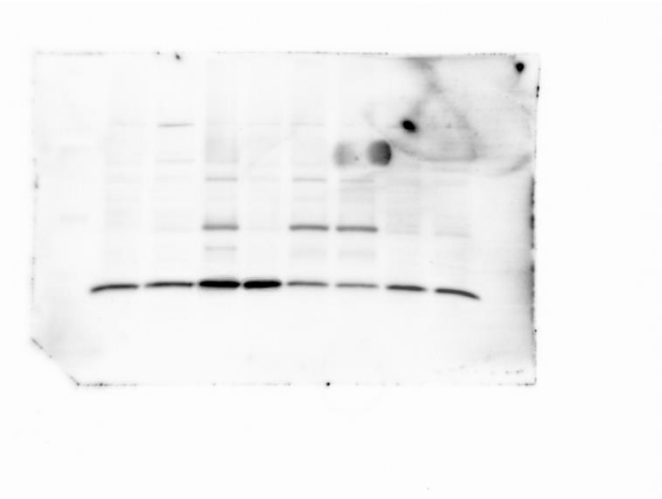

B-actin

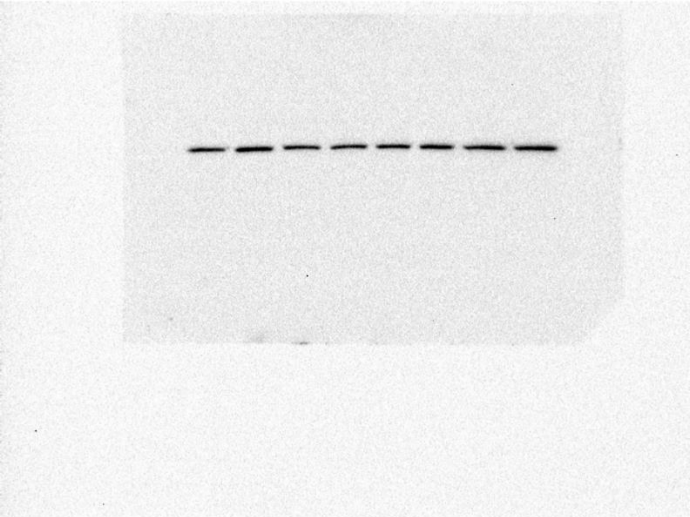

BCL-2

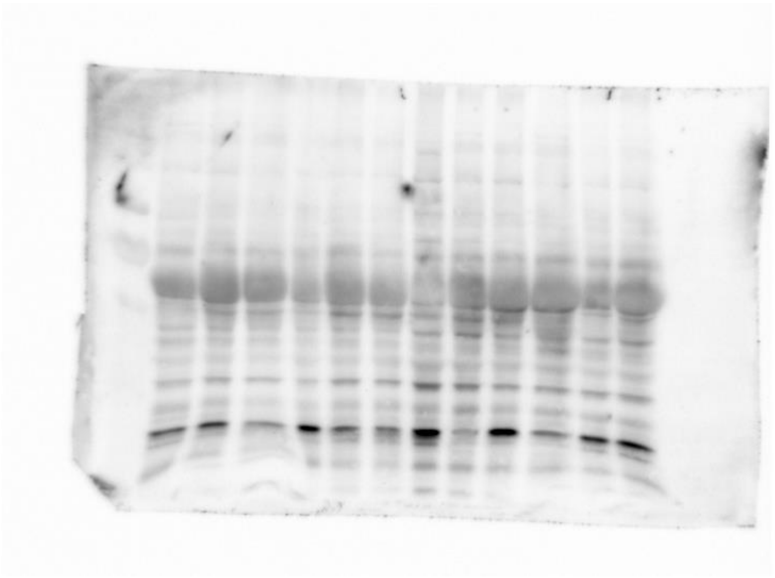

B-actin

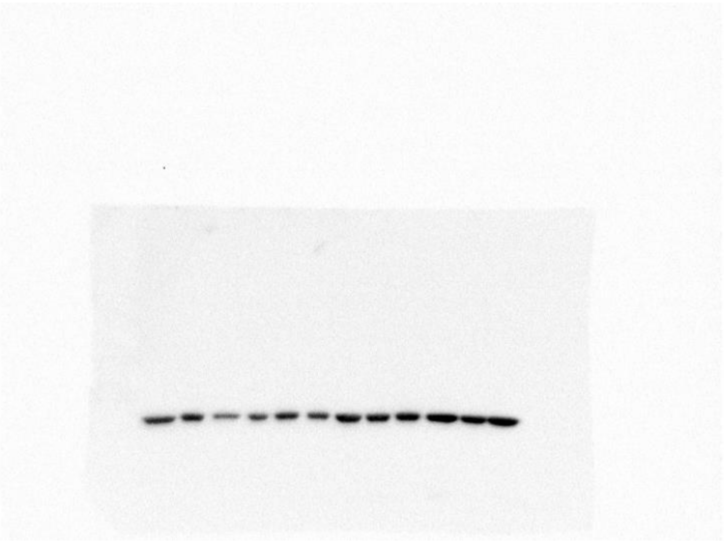

Caspase 9

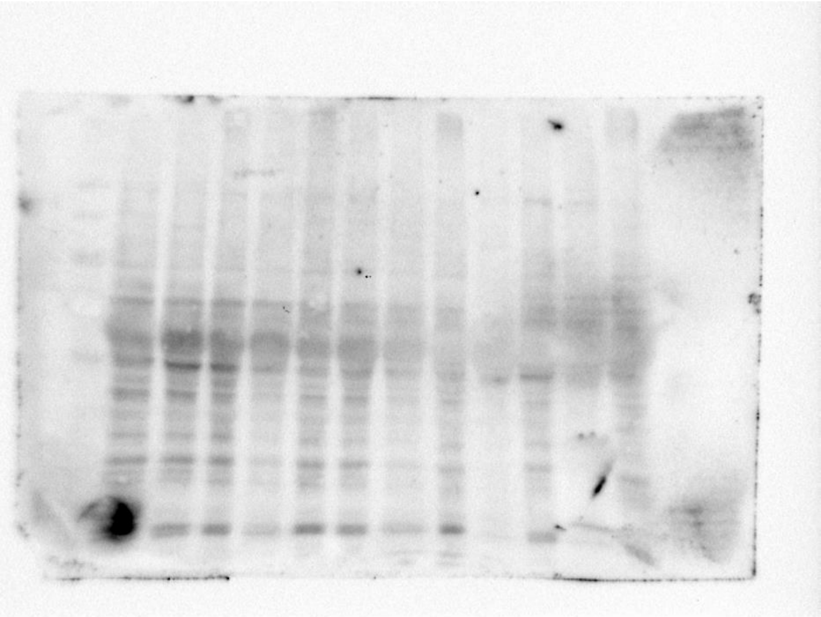

B-actin

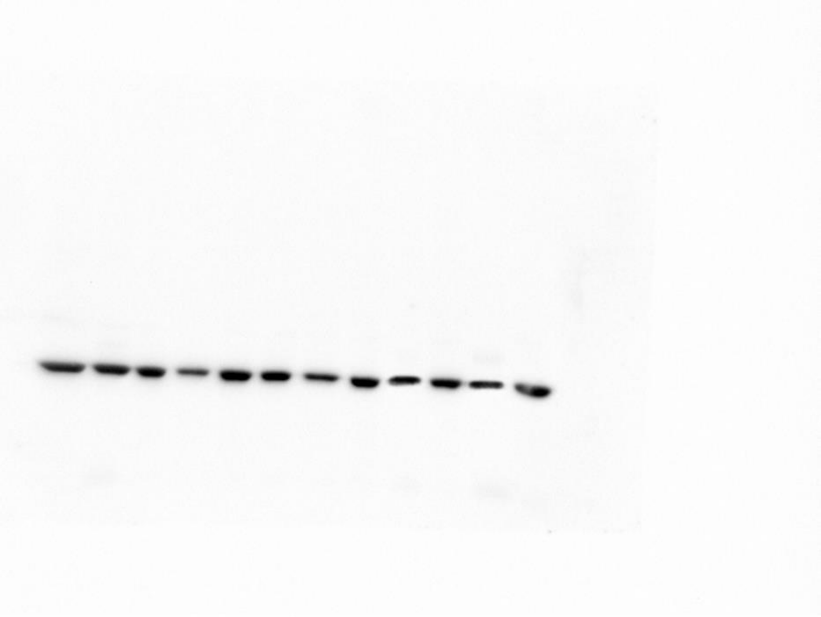

Figure 6.

FOXP3

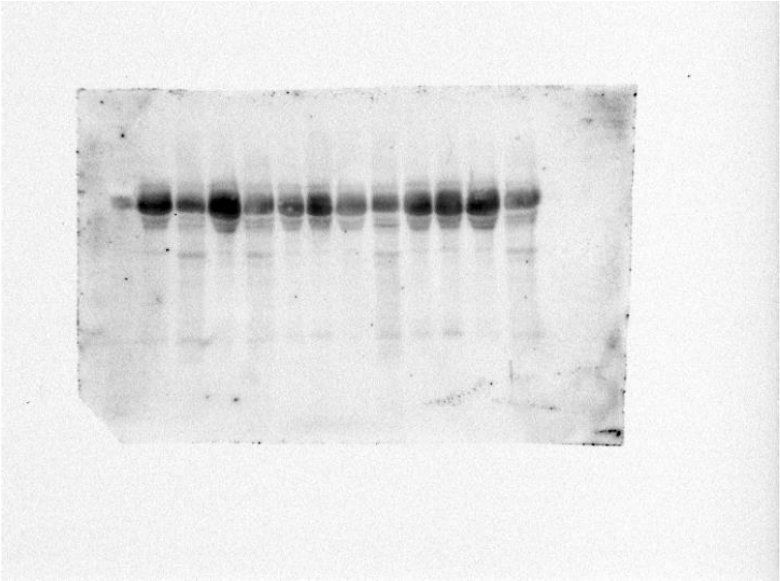

B-actin

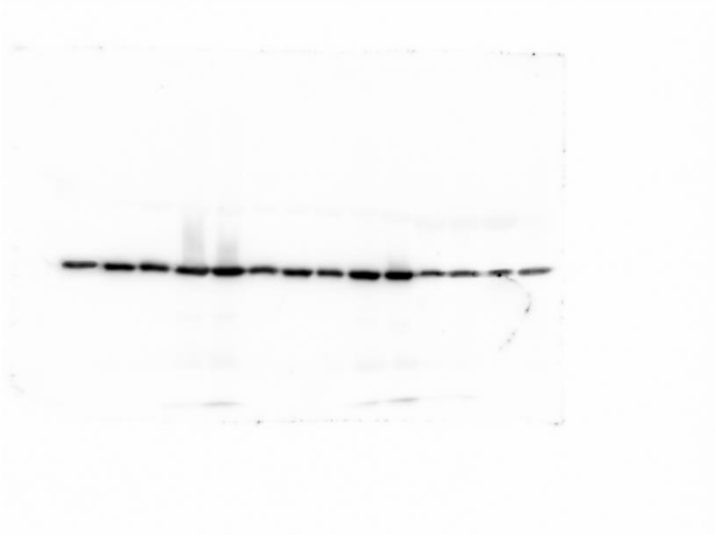

Supplement: Supplementary file 1 — Supplementary Material 1 [file 12958_2024_1246_MOESM1_ESM.pdf]
